# Quantifying invasibility

**DOI:** 10.1111/ele.14031

**Published:** 2022-06-18

**Authors:** Jayant Pande, Yehonatan Tsubery, Nadav M. Shnerb

**Affiliations:** ^1^ Department of Physics Bar‐Ilan University Ramat Gan Israel

**Keywords:** coexistence, community dynamics, demographic stochasticity, environmental stochasticity, extinction risk, invasibility, lottery model, stability, stabilising mechanisms, storage effect

## Abstract

Invasibility, the chance of a population to grow from rarity and become established, plays a fundamental role in population genetics, ecology, epidemiology and evolution. For many decades, the mean growth rate of a species when it is rare has been employed as an invasion criterion. Recent studies show that the mean growth rate fails as a quantitative metric for invasibility, with its magnitude sometimes even increasing while the invasibility decreases. Here we provide two novel formulae, based on the diffusion approximation and a large‐deviations (Wentzel–Kramers–Brillouin) approach, for the chance of invasion given the mean growth and its variance. The first formula has the virtue of simplicity, while the second one holds over a wider parameter range. The efficacy of the formulae, including their accompanying data analysis technique, is demonstrated using synthetic time series generated from canonical models and parameterised with empirical data.

## Graphical abstract

We propose a new formula that quantifies the chance of invasion in fluctuating environments.

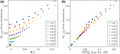

## INTRODUCTION

The capacity of natural systems to maintain diversity (e.g. genetic polymorphism and coexistence of multiple competing species) poses a major challenge to the theory of population genetics and community ecology (Chesson, 2000; Dempster, 1955). Interspecific interactions in diverse communities reflect many complex factors, and their effects are intertwined with strong fluctuations caused by environmental and demographic stochasticity (Kalyuzhny et al., 2014; Lande et al., 2003). A generic method that provides a simple metric to quantify persistence properties is therefore highly desirable.

Turelli's (1978, 1981) approach to this problem focuses on mutual invasibility, that is, on the ‘conditions under which a rare invading species will tend to increase when faced with an array of resident competitors in a fluctuating environment’. This approach greatly simplifies the analysis of the complicated dynamics of a community by reducing it to a series of single‐species invasion problems. All complex interactions are encapsulated in a few effective parameters that reflect the overall influence of the resident species and the environmental fluctuations on a given rare population. The rarity of the focal species allows one to neglect nonlinear (density dependent) effects, facilitating the analysis even further. An analogous approach is taken in permanence (uniform persistence) theories (Hofbauer, 1981; Hutson & Schmitt, 1992; Schreiber et al., 2011; Schuster et al., 1979).

Chesson's modern coexistence theory (Barabás et al., 2018; Chesson, 1982, 1994, 2000; Chesson & Warner, 1981) bases its invasibility analysis on a single parameter, the mean growth rate of the invading species $E\left[r\right]$ (Chesson, 1994; Lewontin & Cohen, 1969; Turelli, 1978). The mean is taken over all the instantaneous growth rates $r\left(t\right)$ where t denotes time. The same parameter, $E\left[r\right]$, is employed in the adaptive dynamics theory of evolution (Brännström et al., 2013; Metz et al., 1995), in the study of epidemics and in many other fields [see Table 1 of Grainger et al. (2019)]. Consequently, ecologists have developed a collection of techniques to infer $E\left[r\right]$ from empirical time series or from models parameterised using empirical data (Grainger et al., 2019). Many contemporary studies of species coexistence and the maintenance of biodiversity analyse this ‘invasion criterion’ $E\left[r\right]$ (Chesson, 2008; Grainger et al., 2019) and partition it between underlying mechanisms such as niche differentiation, fitness differences and so on (Ellner et al., 2016, 2019; Letten et al., 2018). Some authors employ $E\left[r\right]$‐based criteria for mutual invasibility as a metric for stability in empirical studies (Usinowicz et al., 2012, 2017).

However, recent work has revealed that $E\left[r\right]$ is not a reliable *quantitative* indicator of invasibility (Pande et al., 2020a). Systems with different underlying parameters may yield different probabilities of invasion for a species despite having the same numerical value of $E\left[r\right]$. Even worse, invasibility may decrease even as $E\left[r\right]$ increases. Although $E\left[r\right]$ provides a fair *binary* classification—when its value is positive (negative), the extinction state is a repeller (attractor), from which important asymptotic predictions follow (Chesson, 1982, 1994; Ellner et al., 2020; Hofbauer, 1981; Schreiber et al., 2011)—it does not reliably *measure* invasibility.

The failure of $E\left[r\right]$ as a metric reflects two problems (Ellner et al., 2020; Pande et al., 2020a; Pande et al., 2020b). First, since the growth rate r measures the logarithm of abundance ratios, zero‐abundance (extinction) states lead to infinite negative contributions. To avoid this problem, $E\left[r\right]$‐based analyses must neglect demographic stochasticity, the intrinsic stochasticity arising from the birth and death of discrete individuals, despite its crucial importance when the invading population is small. Second (Szilágyi & Meszéna, 2010), $E\left[r\right]$ does not fully include the effect of environmentally induced variation in abundance over time—that is, environmental stochasticity—which affects entire populations collectively.

Thus, given the limitations of $E\left[r\right]$, a better metric is required. Following former studies (Lande et al., 2003), Dean and Shnerb (2020) suggested such a metric for systems with environmental stochasticity. However, they did not take demographic stochasticity into account, and assumed that environmental fluctuations are weak such that the diffusion approximation (Crow & Kimura, 1970; Karlin & Taylor, 1981) is valid.

To provide a more general metric, here we present two new formulae that predict, quantitatively, the chance of invasion. The first of these is based on the diffusion approximation, differing from the work of Dean and Shnerb (2020) in its inclusion of demographic stochasticity. As is usual with the diffusion approximation, this formula is valid only for small levels of stochasticity. Our second formula is based on the large‐deviations WKB (Wentzel–Kramers–Brillouin) approximation, and works for weak as well as strong levels of both demographic and environmental stochasticity. The WKB approach leads to more accurate results over a wide parameter range, and we recommend its use except in cases of very weak stochasticity, where the diffusion approximation formula may work better.

Moreover, our WKB formula converges to the known classical results for establishment probability (Gillespie, 2004; Haldane, 1927; Kimura, 1962) when the environment is fixed through time, and to the expression suggested in Dean and Shnerb (2020) when the diffusion approximation holds. Additionally, our analysis clarifies how the effect of demographic stochasticity may be taken into account indirectly by introducing an ‘extinction threshold’ at the right density. This allows one to extract quantitative predictions from infinite population models, like those used in permanence (uniform persistence) theories (Hofbauer, 1981; Hutson & Schmitt, 1992; Schreiber et al., 2011; Schuster et al., 1979).

Below we derive our two formulae, discuss their accuracy, explain how to extract the parameters required for their use from abundance time series, and finally compare their predictions to simulations of a few canonical models.

## DERIVATION OF INVASION FORMULAE

### The backward Kolmogorov equation and the diffusion approximation

In a community of N individuals, we define the chance of a species of n individuals to grow in abundance and reach a target abundance $n_{f}\ll N$ as $\Pi_{n\to n_{f}}$ (or, in shorter notation, $\Pi_{n}$). $\Pi_{n}$ satisfies the following backward Kolmogorov equation,

(1) $$\Pi_{n}=\sum_{\Delta n=-n}^{N-n}W_{n\to n+\Delta n}\Pi_{n+\Delta n},$$

where $W_{n\to n+\Delta n}$ are the transition probabilities from n to n+Δn individuals during a given period of time τ. This equation reflects the fact that the chance of a population of abundance n to ultimately reach a target abundance $n_{f}$ is equal to the chance to jump from n to any other destination, $W_{n\to n+\Delta n}$, multiplied by the chance to reach $n_{f}$ from this destination. Normalisation requires $\sum_{\Delta n}W_{n\to n+\Delta n}=1$, which means $\Pi_{n}=\text{constant}$ is always a solution of Equation (1). The lower bound Δn=−n corresponds to extinction and the upper bound Δn=N−n to ultimate fixation. The boundary conditions are $\Pi_{0}=0$ (reflecting no chance of invasion from the extinction state) and $\Pi_{n_{f}}=1$ (because $n\geq n_{f}$ implies successful invasion).

In Equation (1), $W_{n\to n+\Delta n}$ are time‐averaged transition probabilities, with the average taken over all the states of the environment. This averaging assumes the time period τ, unspecified so far, to be larger than or equal to the dwell time of the environment (the typical duration for which the environment stays constant before changing), otherwise the transition probabilities are correlated. From here on we assume τ to be the dwell time. We also assume that the typical time to invasion is much larger than τ; in the opposite case the environment varies only slightly over the time required for the population to reach $n_{f}$ (Cvijović et al., 2015; Mustonen & Lässig, 2008) and the chance of invasion may be found by averaging over fixed environments, as explained in the discussion section.

The diffusion approximation (Karlin & Taylor, 1981) replaces the complicated difference Equation (1) by the following simpler differential equation that regards $\Pi_{n}$ as a continuous function of n (writing it as $\Pi \left(n\right)$; throughout this paper, discrete arguments of functions are written as subscripts and continuous ones are put in parentheses),

(2) $$E\left[\Delta n\right]\Pi^{\prime }\left(n\right)+\frac{\mathrm{Var}\left[\Delta n\right]}{2}{\Pi^{\prime }}^{\prime }\left(n\right)=0,\;\Pi \left(0\right)=0,\;\Pi \left(n_{f}\right)=1.$$

Here primes represent derivatives with respect to n. $E\left[\Delta n\right]$ is the mean of the displacement Δn during the time τ, equalling $\sum_{\Delta n}W_{n\to n+\Delta n}\Delta n$. $\mathrm{Var}\left[\Delta n\right]$ is the variance of Δn during the period τ. Both $E\left[\Delta n\right]$ and $\mathrm{Var}\left[\Delta n\right]$ may depend on n.

In the invasion regime, the invading population is rare, so we can neglect density‐dependent effects: different individuals of the invading species do not interact with each other, and the reproductive success of an invader, its chance to produce m offspring, is dictated solely by the environment (including abiotic factors and the effect of the resident species). The total growth in the entire invading population is thus n times $E\left[m\right]$, the expected net growth associated with a single individual, that is, $E\left[\Delta n\right]=E\left[m\right]n$. The variance of this quantity is made up of two terms: demographic stochasticity, which is proportional to n and appears even if the environment is fixed, and environmental stochasticity, which is proportional to $n^{2}$ and appears only when the environment fluctuates (Lande et al., 2003). We employ ℰ, $V_{d}$ and $V_{e}$ as the relevant constants of proportionality, defined by

(3) $$E\left[\Delta n\right]=ℰn,\;\mathrm{Var}\left[\Delta n\right]=V_{d}n+V_{e}n^{2}.$$

Putting the forms of $E\left[\Delta n\right]$ and $\mathrm{Var}\left[\Delta n\right]$ from Equation (3) in Equation (2), the two independent solutions of the differential equation are found to be.

(4) $$\Pi_{I}\left(n\right)=\text{constant},\;\Pi_{\mathrm{II}}\left(n\right)={\left(1+nℛ\right)}^{Q},$$

where

(5) $$ℛ=V_{e}/V_{d},\;Q=1-2ℰ/V_{e}.$$

The boundary conditions $\Pi \left(0\right)=0$ and $\Pi \left(n_{f}\right)=1$ must be satisfied by a linear combination of $\Pi_{I}\left(n\right)$ and $\Pi_{\mathrm{II}}\left(n\right)$, and this yields (see Supplement S1) the invasion formula under the diffusion approximation (DA),

(6) $$\Pi_{n\to n_{f}}^{\mathrm{DA}}=\frac{1-{\left(1+nℛ\right)}^{Q}}{1-{\left(1+n_{f}ℛ\right)}^{Q}}.$$

### Beyond the diffusion approximation: A large‐deviations (WKB) formula

The diffusion approximation is based on two assumptions. It requires Δn to be small with respect to n (so $\Pi \left(n+\Delta n\right)$ is approximated by terms up to ${\left(\Delta n\right)}^{2}$ in a Taylor series), and it assumes that the mean displacement in each step is negligible with respect to its standard deviation (technically, $n{ℰ}^{2}\ll V_{d}+nV_{e}$) (Karlin & Taylor, 1981). When abundance fluctuations and/or ℰ are large, as occurs in many biological scenarios, the diffusion approximation may fail. To overcome this difficulty, it is necessary to take recourse to a large‐deviations technique. In recent years, such techniques, based often on the WKB (Wentzel–Kramers–Brillouin) approximation, have been used in several studies (Assaf & Meerson, 2017; Kamenev et al., 2008; Kessler & Shnerb, 2007; Ovaskainen & Meerson, 2010). Here we employ the WKB technique with the two‐destination approximation (Pande & Shnerb, 2020) scheme that facilitates the analysis and allows us to derive the required invasion formula.

In this analysis, it is more convenient to use the logarithmic parameters z≡lnn and $\Delta z\equiv \ln\left(n+\Delta n\right)-\ln\;n$. On the logarithmic axis, Equation (1) becomes

(7) $$\Pi_{z}=\sum_{\Delta z}W_{z\to z+\Delta z}\Pi_{z+\Delta z},$$

where the bounds of the sum are simply those of Equation (1) transformed to the z‐space. Below we switch between the n‐ and z‐notations or even mix them as convenient, bearing in mind the reciprocal relations z=lnn and $n=\exp\left(z\right)$.

To use the WKB technique, it is helpful to simplify Equation (7) while retaining its character as a difference equation. Our strategy for doing this employs the two‐destination approximation (Pande & Shnerb, 2020), wherein we replace the actual random process in Equation (7) by a simpler process that allows, for each initial location z, only two values for the destination z+Δz. Despite this drastic reduction in the process complexity, no significant errors arise in the dynamical quantities of interest, as long as the first two moments of each jump are preserved (Pande & Shnerb, 2020). The first moment is the mean jump size $E\left[\Delta z\right]$ during the dwell time τ, and the second moment is $E\left[{\left(\Delta z\right)}^{2}\right]$. Note that $E\left[\Delta z\right]$ is related to the invasion growth rate by the relation $E\left[r\right]=E\left[\Delta z\right]/\tau$ (Chesson, 1982).

Here we stipulate that the two possible destinations lie on equal distances to the right and left of the initial position z, though the probability of jumping to the right and to the left may differ (see Supplement S2 for alternative formulations). Under this scheme, which treats Π as a continuous function of z, Equation (7) yields the simpler equation

(8) $$\Pi \left(z\right)=\alpha \Pi \left(z+\beta \right)+\left(1-\alpha \right)\Pi \left(z-\beta \right),$$

where β is the jump size in z‐space and α is the forward jump probability. β and α are in general functions of z, and must satisfy the following relations to leave the first two moments unchanged,

(9) $$\begin{array}{r} \alpha \beta -\left(1-\alpha \right)\beta =\left(2\alpha -1\right)\beta =E\left[\Delta z\right],\\ {\alpha \beta }^{2}+\left(1-\alpha \right)\beta^{2}=\beta^{2}=E\left[{\left(\Delta z\right)}^{2}\right]. \end{array}$$

Here the mean jump in the two‐destination process is found by summing the product of the forward jump (β) and the forward jump probability (α) with the product of the backward jump (−β) and the backward jump probability (1−α), and similarly for the second moment of the jump. Equations (9) imply

(10) $$\beta =\sqrt{E\left[{\left(\Delta z\right)}^{2}\right]},\;\alpha =\frac{1}{2}+\frac{E\left[\Delta z\right]}{2\sqrt{E\left[{\left(\Delta z\right)}^{2}\right]}}.$$

To solve for $\Pi \left(z\right)$ in this two‐destination process, we apply our WKB‐based approach. The basic idea is simple: the diffusion approximation requires $\Pi \left(z\right)$ to be smooth over z, so one may expand $\Pi \left(z+\Delta z\right)$ in a Taylor series and keep only the lowest orders. The large‐deviations technique is based on a less restrictive assumption, that the *logarithm* of Π, that is, $S\left(z\right)\equiv \ln\;\Pi \left(z\right)$, is smooth over z. Therefore, we express $\Pi \left(z\right)$ as $e^{S\left(z\right)}$, and expand $S\left(z+\Delta z\right)$ as $S\left(z+\Delta z\right)\approx S\left(z\right)+\Delta zS^{\prime }\left(z\right)$, where a prime denotes a derivative with respect to z. For brevity of notation, we define $q\equiv S^{\prime }\left(z\right)$. Equation (8) then gives

(11) $$\begin{array}{l} \;e^{S}=\alpha e^{S+\mathrm{q\beta}}+\left(1-\alpha \right)e^{S-\mathrm{q\beta}}\\ \Rightarrow 1=2\alpha \sinh\left(\mathrm{q\beta}\right)+e^{-\mathrm{q\beta}}, \end{array}$$

where sinh is the hyperbolic sine function. This equation has two solutions. The trivial one, q=0, yields the trivial constant solution $\Pi_{I}$. The nontrivial solution for q, given by

(12) $$q=\frac{\ln\left(\frac{1-\alpha }{\alpha }\right)}{\beta },$$

determines the nontrivial solution $\Pi_{\mathrm{II}}\left(z\right)=\exp\left[S\left(z\right)\right]=\exp\left[\int q\left(z\right)\;\mathrm{dz}\right]$. A linear combination of these two solutions must satisfy the appropriate boundary conditions.

Since α and β are both z dependent, it is difficult to integrate $q\left(z\right)$ over z. To obtain an analytic expression, we therefore interpolate between two limits: the large‐n (outer) region where demographic stochasticity is negligible and q is constant, and the small‐n (inner) region where environmental stochasticity is negligible.

**The outer regime**: We have everywhere assumed the invading population to be small (n≪N) such that there are no density‐dependent effects and the only influence of n on the displacement Δz is through demographic stochasticity. Therefore, if n is large enough such that demographic stochasticity is negligible—this condition *defining* the outer regime—Δz is z‐independent and we can define the constants $E_{0}=E\left[\Delta z\right]$ and $V_{e}\equiv E\left[{\left(\Delta z\right)}^{2}\right]-E_{0}^{2}$. The nontrivial solution $\Pi_{\mathrm{II}}^{\text{out}}\left(z\right)$ is, thus,
(13)

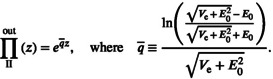

Here $\bar{q}$ is obtained by substituting the expressions for α and β from Equation (10) in Equation (12).

**The inner regime**: If the invading population is very small, birth–death events (among the invaders) are rare, and the environment changes a few times between two successive such events. Since the birth of different individuals in a species takes place in different environments, there are no species‐wide, collective responses to the environment. Accordingly, although environmental variations can still affect the *mean* growth in the abundance (such as through the storage mechanism; Chesson & Warner, 1981; Chesson, 2000), their effect on random fluctuations in the abundance is inherently demographic in nature. The net effect is that in the inner regime the dynamics are those of a population in a constant environment, characterised by the mean growth rate $E_{0}$ and the strength of demographic stochasticity $V_{d}$.

For such a system, with an effectively constant environment, the diffusion approximation is known to be highly accurate (Parsons et al., 2010). In the n‐space, the demographic stochasticity enters Equation (2) (the equation governing the dynamics under the diffusion approximation) only in the second term, $\left(\mathrm{Var}\left[\Delta n\right]/2\right)\Pi^{\prime \prime }\left(n\right)$. Substituting $\mathrm{Var}\left[\Delta n\right]=V_{d}n$, this second term takes the form,

(14) $$\frac{\mathrm{Var}\left[\Delta n\right]}{2}\frac{\partial^{2}\Pi \left(n\right)}{\partial n^{2}}=\frac{V_{d}n}{2}\frac{\partial^{2}\Pi \left(n\right)}{\partial n^{2}}=\frac{V_{d}e^{-z}}{2}\left(\frac{\partial^{2}\Pi \left(z\right)}{\partial z^{2}}-\frac{\partial \Pi \left(z\right)}{\partial z}\right),$$

where the last equation is derived using z=lnn and the chain rule. Combining this term with the drift term, $E_{0}\partial \Pi /\partial z$, we get the diffusion approximation equation valid in z‐space,

(15) $$\left(E_{0}-\frac{V_{d}e^{-z}}{2}\right)\frac{\partial \Pi \left(z\right)}{\partial z}+\frac{V_{d}e^{-z}}{2}\frac{\partial^{2}\Pi \left(z\right)}{\partial z^{2}}=0,$$

where $E_{0}$ and $V_{d}$ are z‐independent constants. Again, there are two solutions to Equation (15), the trivial solution $\Pi_{I}\left(z\right)=\text{constant}$ and the nontrivial solution

(16) $$\Pi_{\mathrm{II}}^{\text{in}}\left(z\right)=e^{-2E_{0}e^{z}/V_{d}}=e^{-2E_{0}n/V_{d}}.$$

Because z=lnn, integration over dz equals integration over dn/n, therefore $\prod_{\mathrm{II}}^{\text{in}}\left(z\right)=\exp\left[\int q\left(z\right)\;\mathrm{dz}\right]=\exp\left[\int q_{\text{in}}\left(n\right)/n\;\mathrm{dn}\right]$, where $q_{\text{in}}\left(n\right)\equiv -2E_{0}n/V_{d}$.

**Interpolation:** Equations (13) and (16) give $\Pi_{\mathrm{II}}\left(z\right)$ when n is large and small, respectively. To interpolate between these two solutions, we interpolate between their corresponding values of q, using

(17) $$q_{\text{inter}}\left(n\right)\equiv \frac{-2E_{0}n}{V_{d}-2E_{0}n/\bar{q}}.$$

Clearly, $q_{\text{inter}}\left(n\right)$ approaches $q_{\text{in}}$ at small n and $\bar{q}$ at large n values, as desired. Therefore,

(18) $$\Pi_{\mathrm{II}}=e^{\int \frac{q_{\text{inter}}}{n}\;\mathrm{dn}}={\left(1-\mathrm{nR}\right)}^{\bar{q}},$$

where

(19) $$R\equiv -\frac{2E_{0}}{\bar{q}V_{d}}.$$

Combining this solution with the trivial (constant) solution so that the boundary conditions ($\Pi_{n=0}=0$ and $\Pi_{n=n_{f}}=1$) are satisfied, we obtain the final WKB‐based invasion formula,

(20) $$\Pi_{n\to n_{f}}^{\mathrm{WKB}}=\frac{1-{\left(1+\mathrm{nR}\right)}^{\bar{q}}}{1-{\left(1+n_{f}R\right)}^{\bar{q}}},$$

with R and $\bar{q}$ defined in Equations (19) and (13).

Importantly, when nR≫1 (which also means $n_{f}R\gg 1$, as $n\leq n_{f}$), the chance of invasion tends to 1 if $\bar{q}<0$ and to ${\left(n/n_{f}\right)}^{\bar{q}}$ if $\bar{q}>0$. Since $\mathrm{sign}\left(\bar{q}\right)=-\mathrm{sign}\left(E_{0}\right)$, when the invading population is large (n≫1/R) and has a fitness advantage ($E_{0}>0$) it is certain to establish. If $n_{f}\gg n$, then the opposite statement also holds: an invading population with negative $E_{0}$ cannot establish. Therefore, only when $1/R\ll n\ll n_{f}$ does invasibility become a binary property for which the mean invasion growth rate $E\left[r\right]$ is a proper metric (Ellner et al., 2020; Pande et al., 2020b).

## INFERRING THE PARAMETERS FROM ABUNDANCE TIME SERIES

Our invasion formulae, Equations (6) and (20), each require three parameters, reflecting the mean and the variance of abundance variations during the dwell time of the environment. Here we explain how to infer these parameters and the value of the dwell time from abundance time series.

We start by considering cases in which artificial time series can be generated using simulations of a community dynamics model parameterised using empirical data. The length and the accuracy of such time series are, in principle, unlimited. Later, we discuss the case of empirical time series, which may be short and plagued by observation errors and thus difficult to accurately estimate the relevant parameters from.

**FIGURE 1:**
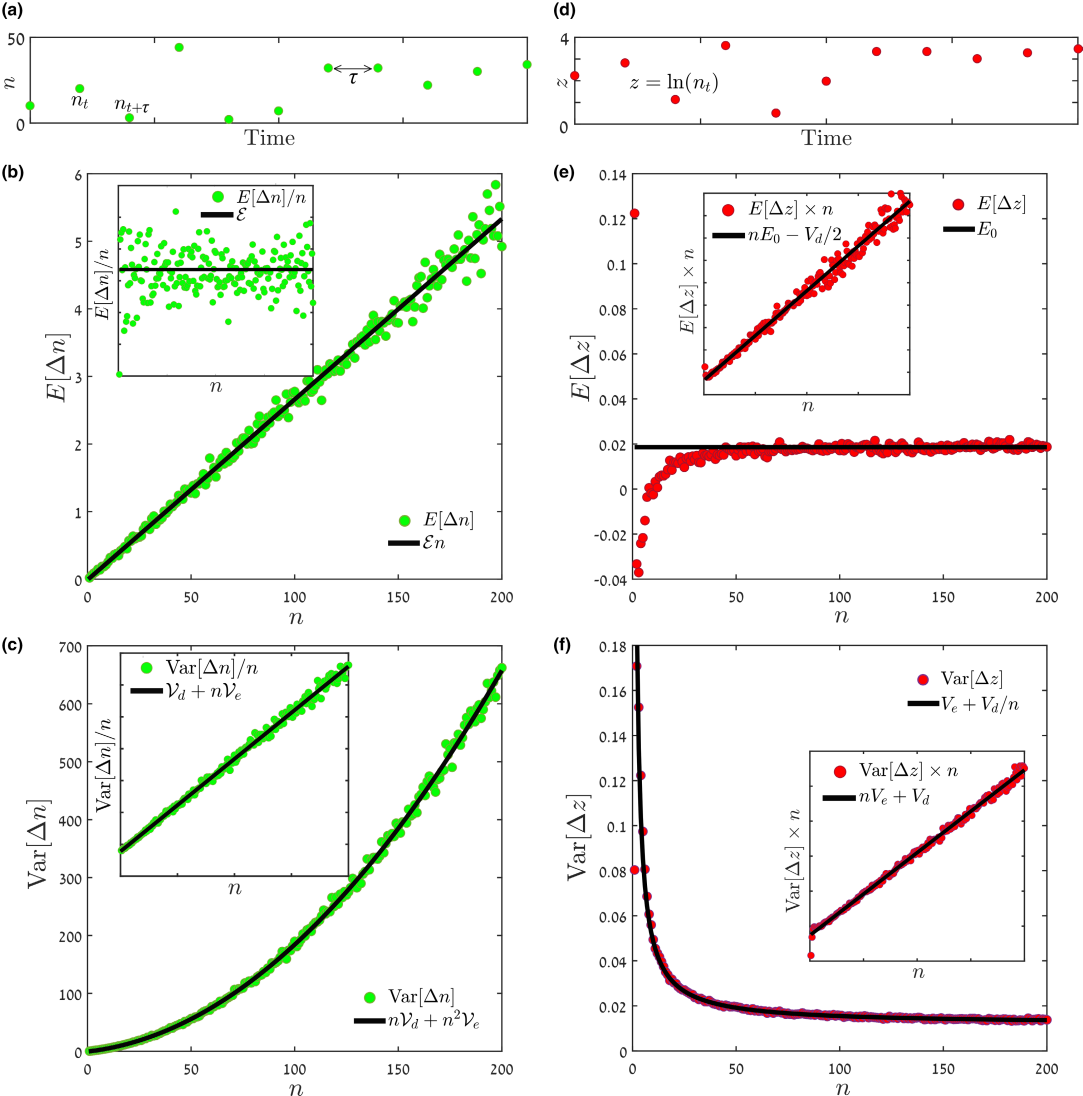
Parameter inference. The raw data is an abundance time series (as obtained from direct observations or a numerical simulation of a properly parameterised model). Here this dataset of $n_{t}$, $n_{t+\tau }$, $n_{t+2\tau }$, … is illustrated in panel (a). Each pair of adjacent points yields a single value $\Delta n=n_{t+\tau }-n_{t}$. The mean value $E\left[\Delta n\right]$ is plotted against n in panel (b). As expected, $E\left[\Delta n\right]$ grows linearly with n, and the constant ℰ is identified from its slope. A graph of $E\left[\Delta n\right]/n$ is shown in the inset, emphasising that this quantity is indeed n‐independent. $\mathrm{Var}\left[\Delta n\right]$ is plotted against n in panel (c). As expected, it takes a quadratic form, $\mathrm{Var}\left[\Delta n\right]=nV_{d}+n^{2}V_{e}$, so $\mathrm{Var}\left[\Delta n\right]/n$ is a straight line whose slope gives $V_{e}$ and the intercept with the vertical axis provides $V_{d}$. An analogous procedure leads to the parameters needed for the WKB‐based formula. The starting point is the time series $z_{t+\tau },z_{t+2\tau },\ldots$, obtained from the time series for $n_{t}$ by the transformation $z_{t}=\ln\;n_{t}$ [panel (d)]. Each pair of points yields one value of Δz, and from these values one calculates $E\left[\Delta z\right]$ [panel (e)]. Note that the parameter $E_{0}$, corresponding to the mean growth rate of a rare population in coexistence theories (more precisely, $E\left[r\right]=E_{0}/\tau$), is the value of $E\left[\Delta z\right]$ when n≫1, that is, when demographic stochasticity is negligible. For smaller values of n, $E\left[\Delta z\right]=E_{0}-V_{d}/\left(2n\right)$, as demonstrated in the inset. To extract the values of $E\left[\Delta z\right]$, we filtered from the dataset all the cases where $n_{t+\tau }=0$; this yields an artificial positive bias when n is very small. Finally, the variance of Δz is shown (in panel (f)) to increase as n decreases, and the inset shows that it satisfies $\mathrm{Var}\left[\Delta z\right]=V_{e}+V_{d}/n$. All the datasets were obtained for the individual‐based version of the lottery model (Chesson, 1982; Chesson & Warner, 1981) with τ=0.2, $s_{0}=0$ and $\sigma_{e}=0.25$ (see Supplement S4 for model details).

The starting point of our analysis is a time series of the abundance $n_{t}$ (Figure 1a). To ascertain the parameters required for the diffusion approximation formula, the mean and the variance of Δn (the change in n during the duration τ) are measured, as in panels (b) and (c), and from these ℰ, $V_{d}$ and $V_{e}$ are determined as illustrated in the insets in these panels. Likewise, to find the parameters for the WKB‐based formula, the abundance series $n_{t}$ is first transformed to a series for $z_{t}=\ln\;n_{t}$ [panel (d)], from which the mean and the variance of Δz are found [panels (e) and (f)], and the parameters $E_{0}$, $V_{d}$ and $V_{e}$ determined [insets in panels (e) and (f)].

Importantly, demographic stochasticity has negligible effect on our ability to measure ℰ. Demographically induced jumps are symmetric in the n‐space, so they leave the value of $E\left[\Delta n\right]$ unchanged. In contrast, demographic stochasticity does diminish the value of $E\left[\Delta z\right]$ (because a symmetric, zero‐mean change in n implies a net negative change in lnn). This effect becomes more pronounced as n decreases, so $E\left[\Delta z\right]$ becomes negative at small values of n [see Equation (15)]. Moreover, because the logarithm of zero diverges, transitions to the state of zero abundance must be excluded from the time series, which generates an artificial bias towards invasion when n is very small. For these two reasons, if the available abundance time series has only a small number of invaders such that demographic stochasticity plays an important role in the dynamics, then the inference of the parameters ℰ, $V_{d}$ and $V_{e}$ from the n‐space analysis is more accurate than the inference of $E_{0}$, $V_{d}$ and $V_{e}$ from the z‐space. In such cases, the diffusion approximation formula Equation (6) may work better than the WKB‐based Equation (20).

**FIGURE 2:**
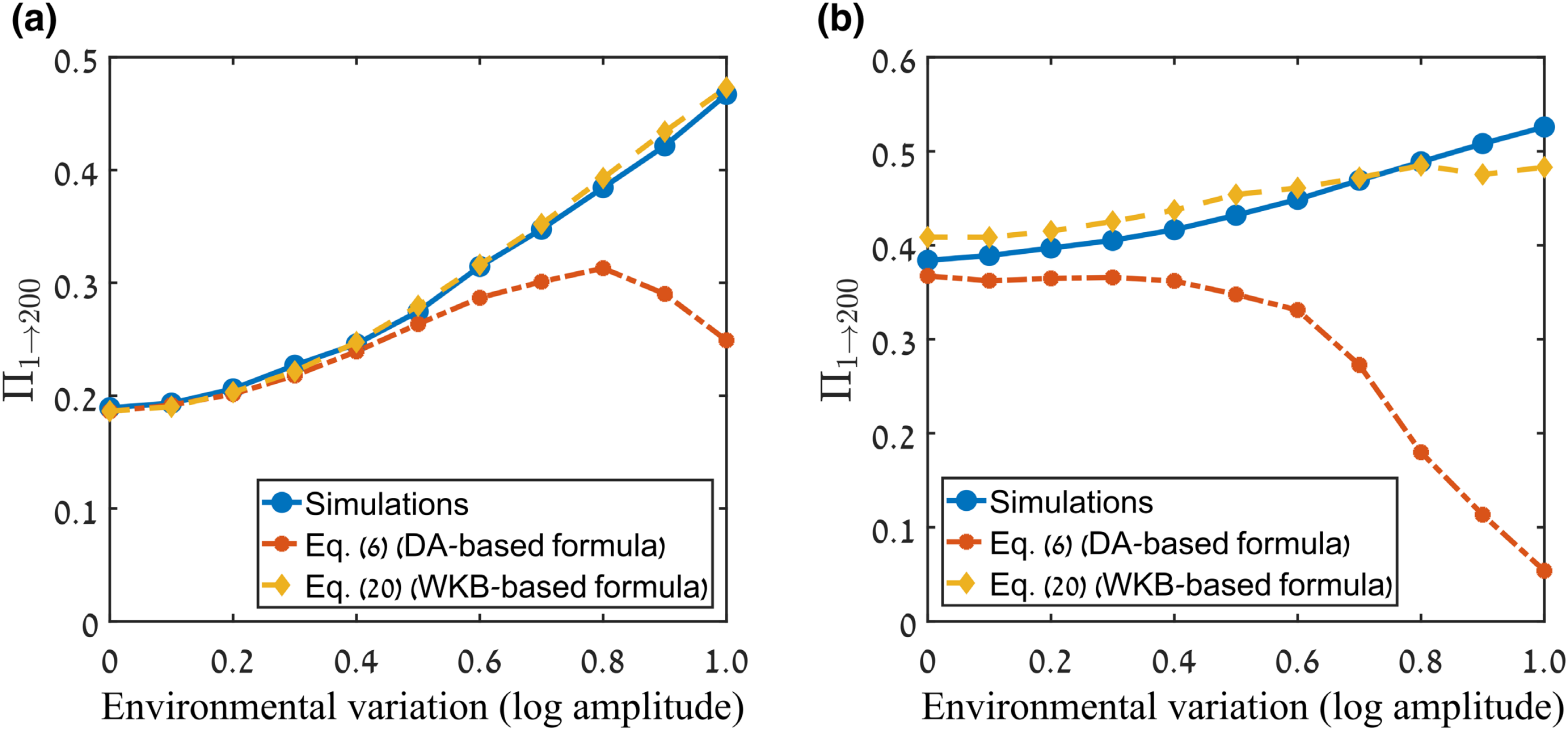
Comparing the formulae. The chance of invasion, $\Pi_{1\to 200}$, as obtained from Monte‐Carlo simulations of the individual‐based lottery model is plotted (blue circles, full lines) against the log amplitude of the environmental variations. These results are compared with the prediction of the diffusion approximation‐based formula, Equation (6) (red circles, dashed‐dotted lines) and the WKB‐based formula, Equation (20) (yellow diamonds, dashed lines), with $n_{0}=1$, $n_{f}=200$. The lottery model parameters were (a) τ=0.1, $s_{0}=0.1$, $N=10^{6}$, and (b) τ=0.3, $s_{0}=0.2$, $N=10^{6}$, where $s_{0}$ indicates the mean fitness advantage of the invading species (see Supplement S4 for model details) and N is the total size of the whole community. The parameters required for the use of each formula were inferred from abundance time series generated using the model, following the procedure described in Figure 1. As is visible, the formula based on the diffusion approximation works well only for low levels of selection and environmental variations. In contrast, the WKB‐based formula agrees with the true chance of invasion for a much wider parameter range. Supplement S5 presents many other plots showing the same comparison for several values of $\tau \in \left[0.1..0.7\right]$ and $s_{0}\in \left[-0.2\ldots 0.4\right]$.

On the other hand, once the relevant parameters are known, the WKB‐based formula performs better than the diffusion approximation‐based one. When the constant selection terms (either ℰ or $E_{0}$) and the environmental stochasticity terms ($V_{e}$ or $V_{e}$) are small, both formulae estimate the chance of invasion well, but in cases of strong selection or large fluctuations the diffusion approximation becomes inaccurate. The superiority of our WKB formula is demonstrated in Figure 2.

The preceding discussion assumes the existence of a long and accurate time series, which is rare when the relevant datasets are empirical. We note nevertheless that the inference of the main parameter used in modern coexistence theory, $E\left[r\right]=E_{0}/\tau$, encounters the same challenges as the inference of the three parameters required for our formulae (see Supplement S3). Moreover, it is often possible to estimate the typical number of propagules per individual, say, or of hatchlings that reach maturity in a nest during a good or a bad year. From such estimates, approximate values for demographic and environmental stochasticity may be determined (as in Sæther et al. (2004), for example), and then our formula may be used to obtain an order of magnitude estimate for the chance of invasion.

Finally, a note on τ, the dwell time of the environment. In our model, the environmental conditions are fixed on average for periods of duration τ and then take an arbitrary and uncorrelated new value. This means that the correlation time of the environment is the mean time before the next shift, that is, τ/2. Therefore, τ equals twice the value of the autocorrelation time of the environment.

## THE INVASION FORMULA IN ACTION

The results shown in Figure 2 (and, more expansively, in Supplement S5) indicate that our WKB‐based formula estimates $\Pi_{n}$ well, and does so for a much wider range of parameters than our diffusion approximation formula. Henceforth, we treat the WKB‐based formula as the recommended one. In this section, we examine its accuracy using a few model systems, and contrast it with quantitative predictions based solely on the invasion growth rate.

We use simulated data from three individual‐based versions of canonical models: the discrete‐time lottery model of Chesson and Warner (1981), its continuous‐time (Moran) analogue, and the Leslie–Gower competition model for trees and saplings employed by Usinowicz et al. (2012) and Usinowicz et al. (2017). Detailed descriptions of these models and of our simulations are provided in Supplement S4. In all these models, there is a negative covariance between environment and competition, and thus environmental stochasticity may promote invasion via the storage mechanism (Chesson, 2000; Chesson & Warner, 1981). This makes the relationship between the underlying process parameters and the invasibility properties intricate and presents a demanding test to our formula.

**FIGURE 3:**
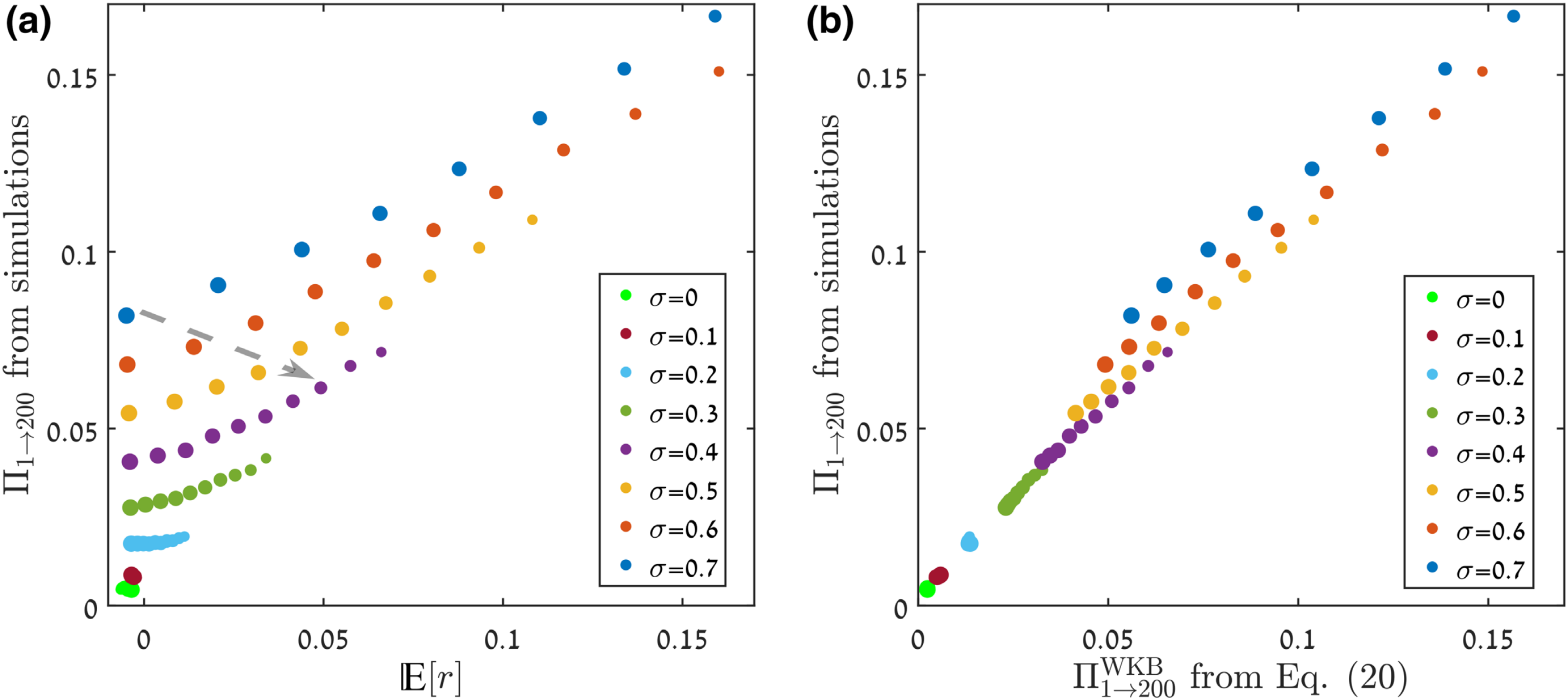
The chance of a single invader (n=1) to reach $n_{f}=200$. This chance was obtained from numerical solution of the discrete‐time lottery model with demographic stochasticity (see Supplement S4) for various values of the model parameters τ (the dwell time) and σ (the amplitude of the temporal fitness variations). The colour of each filled circle indicates the value of σ, while its size is proportional to its τ‐value (between 0.1 and 1). In the left panel, the true chance of invasion is plotted against $E\left[r\right]$, demonstrating that $E\left[r\right]$ is a poor metric: For a given value of $E\left[r\right]$, the true invasibility may have many different values, and when the model parameters τ and σ change (e.g. along the path indicated by the grey arrow), the invasibility may even decrease while $E\left[r\right]$ increases. In the right panel, the true chance of invasion is plotted against the predictions of Equation (20), showing not only a data collapse but also close agreement with the numerical values. All the results were obtained for the case where there is no mean fitness advantage of the invading species, that is, $s_{0}=0$ ($E\left[r\right]$ is positive even in this case due to the storage effect), and with $N=10^{5}$. Similar graphs for $n_{f}=500$ and for $n_{f}=20$ are presented and discussed in Supplement S6, with the latter case providing a good example where Equation (6), based on the diffusion approximation, works better than Equation (20) based on WKB, due to higher accuracy of parameter extraction.

Figure 3 compares the accuracy of the parameter $E\left[r\right]=E_{0}/\tau$ and our $\Pi_{n\to n_{f}}^{\mathrm{WKB}}$ in reflecting the true chance of invasion as measured by simulations, for the case of a single individual trying to invade a community in the discrete‐time lottery model. The different points represent different values of the dwell time τ (in the range $\left[0.1\ldots 1\right]$, indicated by circle size) and the amplitude of environmental stochasticity σ (in the range $\left[0\ldots 0.7\right]$, indicated by colour).

Figure 3a highlights the inadequacy of $E\left[r\right]$ as a quantitative indicator of the chance of invasion, since for any given value of $E\left[r\right]$ the true chance of invasion may have a host of different values. More strikingly, it is possible to change the values of τ and σ in such a way that $E\left[r\right]$ increases even though the true chance of invasion decreases, such as along the trajectory marked by the grey arrow. This shows that even qualitatively a change in $E\left[r\right]$ can fail to reflect the true trend in the invasibility.

In contrast, $\Pi_{n\to n_{f}}^{\mathrm{WKB}}$ reflects faithfully the true chance of invasion, qualitatively as well as quantitatively. This is seen by the fact that the points in panel (b) in Figure 3 lie on a straight line, of slope 45 degrees, showing that the horizontal and vertical values corresponding to a point match—in other words, the predicted chance of invasion (on the horizontal axis) equals, within small error, the measured chance of invasion (on the vertical axis), for all the values of τ and σ probed. The fact that the data collapse on to a line also means that in this case one cannot choose a trajectory along which the predicted and observed values of the invasibility have different trends.

**FIGURE 4:**
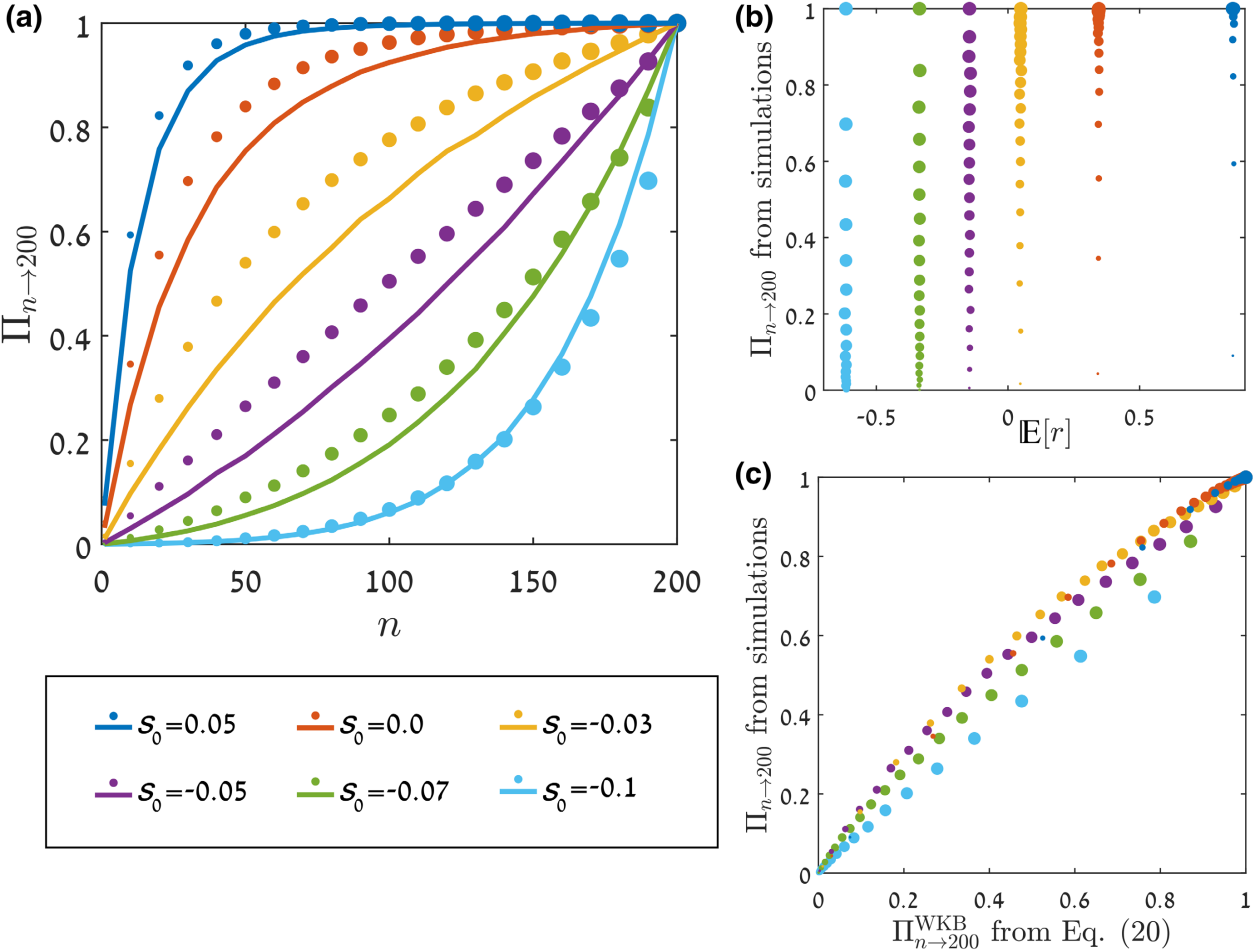
For the continuous‐time (Moran) version of the lottery model, the chance to reach $n_{f}=200$ starting from varying n, as obtained from Monte Carlo simulations (circles), is compared, in panel (a), with the predictions of $\Pi_{n\to n_{f}}^{\mathrm{WKB}}$ from Equation (20) (full lines) for different values of $s_{0}$, the mean selection parameter. In panel (b), the chance of invasion as found from the simulations is plotted against $E\left[r\right]$, with the colour of the points corresponding to their value of $s_{0}$, and their size to the value of n. In panel (c), the same chance of invasion is plotted against $\Pi_{n\to n_{f}}^{\mathrm{WKB}}$. As in Figure 3, all the observed (in simulations) data points collapse on a line when plotted against Equation (20). Other parameters are σ=0.3, τ=0.1 and N=5000. Similar graphs for different values of N are presented in Supplement S7. Even when $s_{0}$ is negative, the curves in panel (a) may have the concave shape associated with a beneficial mutant. This happens because the storage effect (Chesson & Warner, 1981; Dean & Shnerb, 2020) may support invasion by an inferior species.

Figure 4 presents a similar picture for the continuous‐time (Moran) version of the lottery model. Here, the simulations measure the chance of a group of n individuals to establish, with n varying. We allow the mean fitness of the invading species to differ from the mean fitness of the resident species, a property quantified by the parameter $s_{0}$. Again, $E\left[r\right]$ performs poorly as a quantitative predictor for the chance of invasion, whereas $\Pi_{n\to n_{f}}^{\mathrm{WKB}}$ predicts the true invasibility well. The limitations in $E\left[r\right]$ are more prominent here, since $E\left[r\right]$ is by definition oblivious to n, so all points for a given value of $s_{0}$ have the same value of $E\left[r\right]$ irrespective of n, despite being widely separated in their values of the observed invasibility.

**FIGURE 5:**
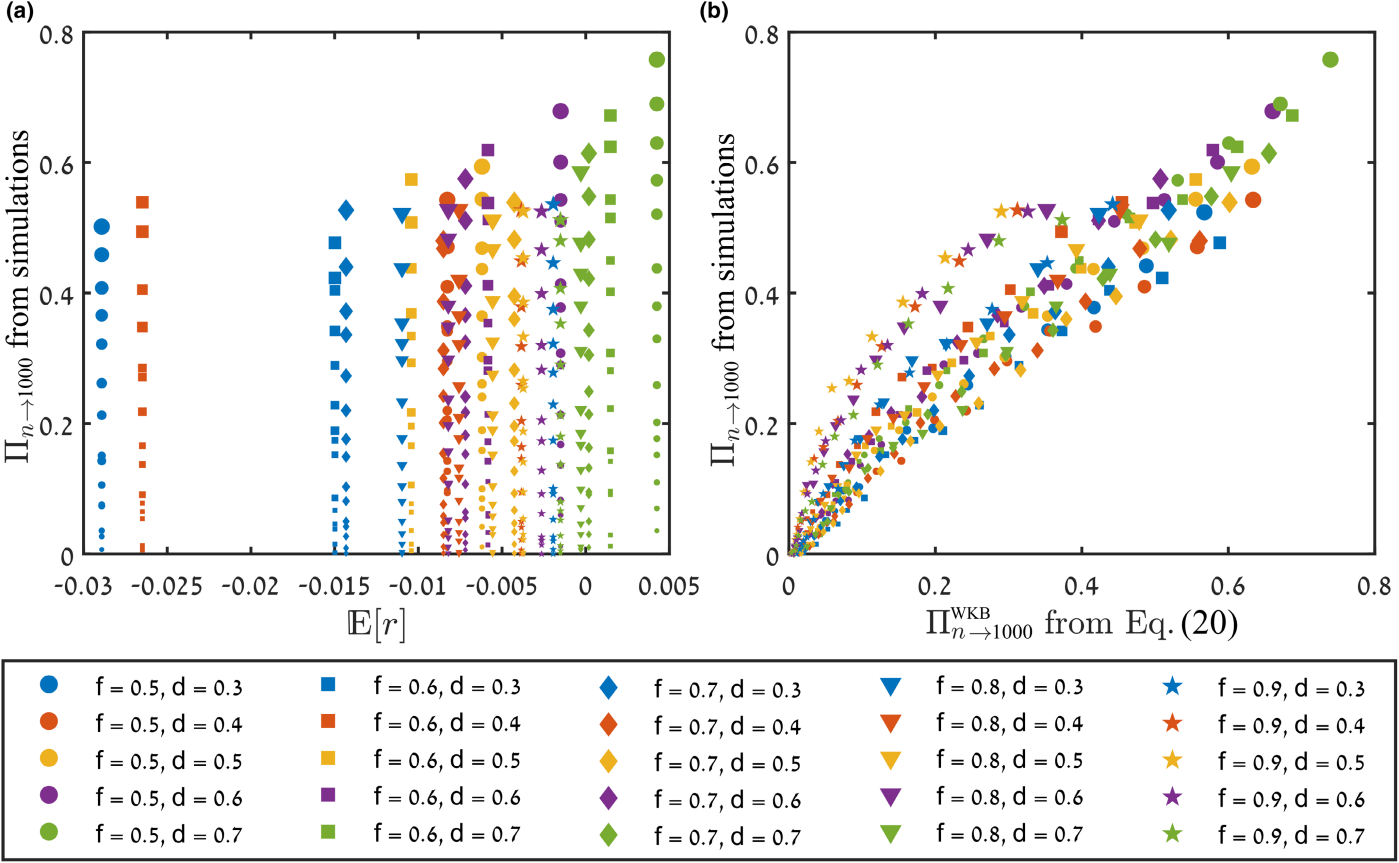
Chance of invasion in the Leslie–Gower forest dynamics model of Usinowicz et al. (2012) and Usinowicz et al. (2017), as described in Supplement S4. For a community size of N=10000, we calculated the chance to reach $n_{f}=1000$, starting from varying n, and plotted the results against $E\left[r\right]$ (panel a) and against the predictions of Equation (20) (panel b). The size of each point is proportional to the initial population $n\in \left[1..200\right]$, different colours correspond to different chances of adult tree survival $d\in \left[0.3..0.7\right]$ and different symbols represent different chances of sapling survival $f\in \left[0.5..0.9\right]$. In our simulations, we employed the empirical recruitment rates for *Spondias mombin* and *Spondias radlkoferi*, as provided in Usinowicz et al. (2012), scaled by a constant factor. A step‐by‐step description of the analysis is presented in Supplement S8.

In Figure 5, we show results for the individual‐based version of the Leslie–Gower model for the dynamics of trees and saplings. We have used the recruitment rates for the species *Spondias mombin* and *Spondias radlkoferi* (scaled by a constant factor), as provided in Usinowicz et al. (2012). While in the models used for Figures 3 and 4 the dwell time τ is a model parameter, here it needs to be identified as well from the abundance time series (just like the parameters $E_{0}$, $V_{d}$ and $V_{e}$). As a run‐through of our whole method, we have provided a full step‐by‐step analysis for this model, including the generation of the abundance time series and the identification of all the required parameters, in Supplement S8.

For this model, the two panels in Figure 5, like in Figures 3 and 4, compare the efficacy of $E\left[r\right]$ and $\Pi_{n\to n_{f}}^{\mathrm{WKB}}$ as quantitative indicators of invasibility. Each point represents a different set of sapling survival probability ($f\in \left[0.5\ldots 0.9\right]$, indicated by symbol shape), adult tree survival probability ($d\in \left[0.3\ldots 0.7\right]$, indicated by symbol colour) and initial abundance ($n\in \left[1\ldots 200\right]$, indicated by symbol size). Like in Figure 4, since $E\left[r\right]$ is insensitive to n, there are vertical bands, corresponding to different values of f and d, of constant $E\left[r\right]$ value and widely divergent values of the observed chance of invasion in panel (a). In contrast, the predicted and the observed chances of invasion [shown in panel (b)] agree as the points lie on a straight line of slope 45 degrees, to within experimental error.

## DISCUSSION

The chance of an invading population to grow in abundance to a large size governs many ecological and evolutionary processes, and is one of the main metrics used to assess the stability of an ecological community. In this paper, we have provided formulae that quantify this chance in fluctuating environments in terms of the dwell time of the abundance fluctuations, the mean and the variance of these fluctuations, and the initial and target densities.

In the existing literature, the sign or the numerical value of $E\left[r\right]$ is used as a proxy for the chance of invasion (Ellner et al., 2016, 2019; Letten et al., 2018; Usinowicz et al., 2012, 2017). However, as noted by Grainger et al. (2019), ‘demographic stochasticity can have important consequences for low‐density invasion growth rates, but this is frequently overlooked … the impact of demographic stochasticity on outcomes predicted by ($E\left[r\right]$) remains largely an open question’. As shown above, these are two independent and important problems. First, the estimation of $E\left[r\right]$ from low‐density time series yields reduced values for $E\left[r\right]$ and may even invert its sign (see Figure 1e). Second, even if $E\left[r\right]$ is known, it cannot be used as a quantitative metric unless the invading population is large enough ($1/R\ll n\ll n_{f}$).

The formulae provided here, Equations (6) and (20), facilitate a more informed analysis. First, the invasibility may be calculated using these formulae for any size of the invading population, as our treatment takes demographic stochasticity into account. Second, the diffusion approximation formula Equation (6) depends on the parameters in the n‐space, in particular, on $ℰ=E\left[\Delta n\right]/n$, which is not affected by demographic stochasticity (and does not diverge upon transitions to the zero‐abundance state, unlike $E\left[r\right]$). This formula may thus be used even when the relevant datasets are limited to small abundances. The WKB‐based formula Equation (20) may be employed when $E\left[\Delta z\right]$ is calculated in the regime where it is independent of the abundance.

The price one has to pay for using our formulae is the need to calculate two more quantities, $V_{d}$ and $V_{e}$ (or $V_{d}$ and $V_{e}$). As illustrated in Figure 1, the calculation of these additional parameters is of a similar level of complexity as that of $E\left[r\right]$ (see Supplement S3). In some cases, even when the time series are not long enough, it may be possible to estimate these parameters to within acceptable error through heuristic means, in which case $\Pi_{n\to n_{f}}^{\mathrm{WKB}}$ (or $\Pi_{n\to n_{f}}^{\mathrm{DA}}$) is again a more informative metric of the chance of invasion than $E\left[r\right]$.

Given the simplicity and accuracy of our formulae for $\Pi_{n}$, we suggest that they replace $E\left[r\right]$ as an invasion metric in ecological and evolutionary analysis. A larger $\Pi_{n}$ implies a larger chance for a given population to invade or to recover from disturbances. Therefore, all other things (rate of disturbances, rate of migration from a regional pool, etc.) being equal, an increase in the value of $\Pi_{n}$ implies more successful colonisation and lower extinction rates, unlike the case for $E\left[r\right]$ (Pande et al., 2020a).

As an invasion parameter $\Pi_{n}$ may be used for the same purposes for which $E\left[r\right]$ is currently employed. In particular, one may partition its numerical value to study the contribution of specific mechanisms, like covariance between the competition and the environment (Ellner et al., 2016). Moreover, our formulae allow a quantitative estimate of the invasibility even when a precise numerical assessment through simulations is very difficult or computationally expensive, for instance, when $n_{f}$ is huge or when the chance of invasion is very close to 0 or to 1. This latter case is the situation in Usinowicz et al. (2017), where a comparison between the stability of different tree communities requires one to quantify the invasibility even though it is very close to 1.

Beyond their potential use in coexistence theory and related problems, our formulae provide some important insights.

First, they help to define a value of $n_{f}$, the target abundance at which a species may be said to have established successfully. Two simple alternatives are choosing $n_{f}$ to be some fixed large number (independent of the overall community size) or a fixed frequency (a given fraction of the community), but these choices are somewhat arbitrary. Our WKB formula suggests that a natural definition for $n_{f}$ (when $E\left[r\right]$ is positive, so $\bar{q}$ is negative) is the abundance above which the chance of extinction is small, that is, $n_{f}∼\exp\left(1/|\bar{q}\right)/R$.

Second, because the incorporation of both demographic and environmental stochasticity in a model is a complicated task, many population models, like those used in permanence (uniform persistence) theories (Hofbauer, 1981; Hutson & Schmitt, 1992; Schreiber et al., 2011; Schuster et al., 1979), deal only with environmental variations, whose effect is generally stronger than that of demographic variations (Lande et al., 2003). However, without demographic stochasticity, the system never reaches the zero‐abundance state, so in these theories one must introduce an arbitrary threshold below which a population is considered extinct. In Supplement S9, we show that our formulae converge to the expressions obtained in the literature when demographic stochasticity is neglected, *if* the extinction threshold is chosen at

(21) $$n_{\mathrm{th}}=V_{d}/V_{e},$$

that is, at the abundance level at which the strengths of demographic and environmental stochasticity become equal. This result clarifies the range of parameters for which infinite population models are applicable: the total population has to be much larger than $n_{\mathrm{th}}$. Furthermore, by introducing an absorbing boundary at this threshold value $n_{\mathrm{th}}$, one may obtain quantitative approximations (for quantities like the mean time to extinction) from models that disregard demographic stochasticity.

Our invasion formulae have a few limitations. First, to derive them we assumed that the dwell time τ of the environmental variations is smaller than the time required for a population to successfully invade. When τ is larger than the typical invasion time (the “quenched” scenario of Mustonen & Lässig, 2008), the actual value of τ becomes insignificant, since the environment typically remains fixed throughout the invasion process (see Supplement S6). Our formulae do not apply in this regime, but they are not required either, since in this case one may use the well‐known formula for the probability of invasion in a fixed environment (Haldane, 1927; Kimura, 1962) and determine the overall invasibility by taking an appropriately weighted expectation value over all possible environmental states. This fixed‐environment invasion formula is, by construction, the limit $V_{e}\to 0$ of Equation (20) [and of $V_{e}\to 0$ of Equation (6)].

Second, as mentioned earlier, Equation (20) holds only if the parameters $E\left[\Delta z\right]$, $V_{d}$ and $V_{e}$ are constant in the invasion regime, which requires the ratio $n_{f}/N$ to be small, otherwise density‐dependent effects appear.

Third, to use abundance time series, one must contend with sampling errors, which are common in ecological surveys (Clark & Bjørnstad, 2004). If these errors are unbiased, they do not affect $E\left[r\right]$ (or $E\left[\Delta z\right]$), but they can overestimate $V_{e}$. In some cases, these errors may be filtered out using standard techniques (Clark & Bjørnstad, 2004; Kalyuzhny et al., 2014). Otherwise, an informed guess about their effect on $V_{e}$ is required.

Species richness and genetic polymorphism reflect the balance between the rate of extinction and the rates (of colonisation, mutation, speciation, etc.) at which new types get established in the community. Invasibility, the chance of a population to invade or to recover from low densities, plays a crucial role in both kinds of processes. Consequently, our formulae should facilitate a more accurate and reliable assessment of these important aspects of life science systems.

## AUTHORS’ CONTRIBUTIONS

J.P. and N.M.S. developed the formulae and analysed the data. Y.T. simulated the Leslie‐Gower model for tree‐sapling dynamics.

### PEER REVIEW

The peer review history for this article is available at https://publons.com/publon/10.1111/ele.14031.

## Supporting information


Data S1

## Data Availability

Data (MATLAB code) supporting the results is archived on Figshare at https://doi.org/10.6084/m9.figshare.19746304.v1.
