# Supplementary material for: Quantifying invasibility
Source: Ecol Lett. 2022 Jun 18;25(8):1783–94. doi: 10.1111/ele.14031 (PMC9543749; doi:10.1111/ele.14031)
Supplement: Supplementary file 1 — Data S1 [file ELE-25-1783-s001.pdf]

Supplements for:
Quantifying invasibility

Jayant Pande, Yehonatan Tsubery and Nadav M. Shnerb

Department of Physics, Bar-Ilan University, Ramat-Gan IL52900, Israel

S1. DERIVATION OF EQ. (6) OF THE MAIN TEXT

In Eq. (6) in the main text we presented the invasion formula under the diffusion approximation. Here we show its derivation based on Eqs. (2)-(5).

To be specific, we are looking for a solution for Eq. (2) of the main text, which is

$$\mathbb{E}[\Delta n]\Pi'(n) + \frac{\mathrm{Var}[\Delta n]}{2}\Pi''(n) = 0, \tag{S1}$$

with the boundary conditions

$$\Pi(0) = 0, \quad \Pi(n_\mathrm{f}) = 1. \tag{S2}$$

Since Eq. (S1) is a second-order differential equation, it admits two linearly independent solutions, which we call $\Pi_\mathrm{I}(n)$ and $\Pi_\mathrm{II}(n)$ and which are presented in Eq. (5) in the main text, as

$$\Pi_\mathrm{I}(n) = \mathrm{constant}, \qquad \Pi_\mathrm{II}(n) = (1 + n\mathcal{R})^{\mathcal{Q}}. \tag{S3}$$

The general solution is a linear combination of these two independent solutions, namely,

$$a_1\Pi_\mathrm{I}(n) + a_2\Pi_\mathrm{II}(n), \tag{S4}$$

where a_1 and a_2 are arbitrary constants. To satisfy the boundary conditions, one must choose a_1 and a_2 so that

$$a_1\Pi_\mathrm{I}(n=0) + a_2\Pi_\mathrm{II}(n=0) = 0, \qquad a_1\Pi_\mathrm{I}(n=n_\mathrm{f}) + a_2\Pi_\mathrm{II}(n=n_\mathrm{f}) = 1. \tag{S5}$$

Since $\Pi_\mathrm{I}(n=0)$, $\Pi_\mathrm{II}(n=0)$, $\Pi_\mathrm{I}(n=n_\mathrm{f})$ and $\Pi_\mathrm{II}(n=n_\mathrm{f})$ are all known, we now have two equations from which the two unknown constants a_1 and a_2 can be determined. To do this, we first absorb the constant solution Π_1 in a_1, so that the two equations (S5) become

$$a_1 + a_2 = 0, \qquad a_1 + a_2(1 + n_\mathrm{f}\mathcal{R})^{\mathcal{Q}} = 1. \tag{S6}$$

Solving these we get

$$a_1 = -a_2 = \frac{1}{1 - (1 + n_\mathrm{f}\mathcal{R})^{\mathcal{Q}}}. \tag{S7}$$

Plugging these constants into Eq. (S4), we obtain

$$\Pi^{\mathrm{DA}}_{n\to n_{\mathrm{f}}} = \frac{1-(1+n\mathcal{R})^{\mathcal{Q}}}{1-(1+n_{\mathrm{f}}\mathcal{R})^{\mathcal{Q}}} \ , \tag{S8}$$

which is Eq. (6) of the main text.

S2. A DETAILED DISCUSSION OF THE TWO-DESTINATION APPROXIMATION

In this section we provide more details concerning the derivation of Eq. (20) of the main text, and in particular explain the differences between the two-destination approximation used in [1] and the one used here. To do this we return to the basic ideas in this approximation and highlight the assumptions made, the alternative approaches possible and their relevance to the final outcome.

The analysis of the backward Kolmogorov equation (Eq. (1) of the main text) is simplified if the transition probabilities $W_{n\to n+\Delta n}$ are defined over the dwell time of the environmental fluctuations. This is because in that case the different states of the environment can be averaged over without losing the effect of temporal correlations (which result from adjacent steps tending to be in the same environment). Therefore, in Eqs. (1) and (7) of the main text the transition probabilities are defined accordingly, i.e., as the chance to jump from n to $n+\Delta n$ after τ generations, where τ is the dwell time of the environment (τ generations equal $N\tau$ elementary birth-death events, since one generation is defined as N elementary events). Even with this simplification, however, there remains the problem that a single abundance state n might go to many possible destination states over a τ-interval. This plurality of the destination states renders the analysis complicated.

The "jack-of-all-trades" approach to this problem is the diffusion approximation, which is based on an expansion of the quantity of interest ($\Pi(n+\Delta n)$, say) to second order in Δn. This procedure involves two levels of approximation. The first level is the continuum approximation, which depends on Π_n being a smooth function over the integers. Second, the second-order expansion implies that only the first two moments of $W_{n\to n+\Delta n}$ are important, so the mean and the variance (or the mean and the second moment) of the jump at each n determine the outcome of the process. These steps, in conjunction with the identification of the second moment $\mathbb{E}[(\Delta n)^2]$ with the variance $\mathrm{Var}[\Delta n]$ (which reflects the assumption $\mathcal{E}^2 \ll \mathbb{E}[(\Delta n)^2]$), yield Eq. (2) of the main text.

The failure of the diffusion approximation in some circumstances has been analyzed in many studies [2–4]. In [1] we pointed out that the main problem is with the first assumption, not the second: relying only on the first two moments yields accurate results as long as one avoids the continuum approximation. Therefore, we suggested an approach based on a large-deviations (controlling-factor WKB) technique that resolved the continuum approximation problem by assuming that the logarithm of Π_n, instead of Π_n itself, is smooth. We retained the second assumption, namely that the outcome depends only on the first two moments. This meant that the actual process (like in Eq. (7) of the main text) could be replaced by an effective process in which instead of all the different possible destination states there are only two destinations, properly chosen such that the mean and the second moment of the jump, defined by

$$\mathbb{E}[\Delta z] = \sum_{\Delta z}(\Delta z)W_{z\to z+\Delta z}, \qquad \mathbb{E}[(\Delta z)^2] = \sum_{\Delta z}(\Delta z)^2 W_{z\to z+\Delta z}, \tag{S9}$$

are preserved. Both these quantities are z-dependent, but for the sake of simplicity we omit the z index in this discussion.

There are infinitely many possible choices for this two-destination scheme. In general, for each z one may replace the actual full process by a process with only two jumps, from z to $z + z_1$ with probability α and from z to $z - z_2$ with probability $1 - \alpha$, where the only restriction on z_1, z_2 and α is that the resulting mean and second moment of the new process are the same as those of the original process. The scheme we adopted in [1] was based on equal jump probabilities ($\alpha = 1/2$) and unequal jump lengths ($z_1 \neq z_2$). Here, in contrast, we adopt a different scheme, in which the jump lengths are kept equal in magnitude (but opposite in direction) and the jump probabilities are unequal, which leads to the following choice:

Destination	Probability	Jump increment
$z + \beta$	$\alpha = \frac{1}{2} + \frac{\mathbb{E}[\Delta z]}{2\sqrt{\mathbb{E}[(\Delta z)^2]}}$	β
$z - \beta$	$1 - \alpha = \frac{1}{2} - \frac{\mathbb{E}[\Delta z]}{2\sqrt{\mathbb{E}[(\Delta z)^2]}}$	$-\beta$

where $\beta = \sqrt{\mathbb{E}[(\Delta z)^2]}$. This choice leads to Eq. (11) of the main text for q, with the solution provided in Eq. (12).

This raises the question: what are the differences between the two schemes ("equal chances, unequal jumps" as used in [1], and "equal jumps, unequal chances" as used here)? Is there a "correct" scheme? The following comments serve to answer these questions and to clarify some other aspects of the WKB technique.

1. The scheme used in the current paper is technically more convenient, as it allows an analytical solution for the WKB transcendental equation (Eq. (11) of the main text), whereas the corresponding transcendental equation discussed in [1], when the jumping probabilities are equal and the jumps differ in their length, admits only approximate or numerical solutions.

2. Importantly, the solutions for q for the transcendental equations obtained under the two schemes coincide in the small-q regime, where the mean $\mathbb{E}[\Delta z]$ is much smaller than the second moment $\mathbb{E}[(\Delta z)^2]$, i.e., where stochasticity is strong.

3. The solutions differ significantly from each other in the large-q regime, when the mean value of the jump length is approximately equal to, or larger than, its standard deviation. In this regime the scheme we have used in [1] yields a diverging value for q, while the expression in Eq. (12) is finite.

   The origin of this difference is easy to understand. When one uses the "equal probabilities, unequal jump lengths" scheme, the length of the shorter jump is the mean minus the standard deviation. When the mean is larger than the standard deviation both jumps are in the same direction, so the abundance either grows

in every step or diminishes in every step. Thus the chance of fixation is either strictly 0 or strictly 1, which manifests itself in the divergence of q. In contrast, under the "equal jump lengths, unequal jump probabilities" scheme implemented here, jumps are always in opposite directions and the bias (i.e., $\mathbb{E}[\Delta z]$) affects only their probabilities (the α-values), so for any strength of the bias there is still a chance (even if small) for both extinction and fixation.

Which of the two schemes is more suitable depends on the statistics of the fitness variations. If the possible deviations of fitness values from their mean are limited (in other words, if the probability distribution function from which the fitness fluctuations are drawn is compact), then once $\mathbb{E}[\Delta z]$ gets large enough the actual process (without demographic stochasticity) will move deterministically towards either fixation or extinction. The correct scheme, then, is the one used in [1], i.e., with equal jump probabilities and unequal magnitudes of the jump length, in which q diverges when z_1 and z_2 have opposite signs. In contrast, when fitness fluctuations can grow without bound, even if with low probability (i.e., when the probability distribution function is non-compact, e.g., Gaussian), then the scheme used in the current paper, with equal magnitudes of the jump length and unequal jump probabilities, is better. This is so because in this case there is always a chance for movement in both directions, towards extinction and fixation.

S3. POSSIBLE ISSUES IN USING EMPIRICAL TIME SERIES

Our WKB-based formula Eq. (20) in the main text employs three parameters, $\mathbb{E}[\Delta z]$, $V_{\rm d}$ and $V_{\rm e}$ (similarly, our diffusion approximation-based formula Eq. (6) in the main text employs the three parameters $\mathcal{E}$, $\mathcal{V}_{\rm d}$ and $\mathcal{V}_{\rm e}$). When these parameters are extracted from numerical simulations (which may be parameterized using field observations), as in the examples provided in this paper, the length of the available time series is, in principle, unlimited. The situation changes when one uses empirical time series, which may be rather short. In what follows we provide a few rules of thumb for the required length of these time series.

Let us first address the estimation of $\mathbb{E}[\Delta z]$ (or $\mathbb{E}[r]$). Given a time series of $M+1$ abundance values $\{n_{t=1}, ..., n_{t=M+1}\}$, one may extract M points of Δz values $\{\ln(n_2/n_1), ..., \ln(n_{M+1}/n_M)\}$ (with the corresponding r values obtained by dividing each logarithm by the corresponding time lag). A reliable estimation requires these data points to be independent, so the time lag between each pair of points has to be larger than δ, the dwell time of the environment. Therefore, to accurately estimate $\mathbb{E}[\Delta z]$, it is necessary to first have an estimation of the correlation time of the environment (either from the data or from a priori knowledge of the variability of the relevant environmental factors).

Once a set of M independent points is given, its mean

$$\mu = \frac{1}{M}\sum_{i=1}^{M}(\Delta z)_i$$

provides the best estimation for $\mathbb{E}[\Delta z]$, but the uncertainty of this estimation clearly depends on the variance of the distribution from which the elements of the original time series were picked. In the best-case scenario, when the variance $\sigma^2_{\rm std}$ of the underlying distribution is known precisely ($\sigma_{\rm std}$ being the standard deviation of this distribution) and $(\Delta z)_i$ is, roughly speaking, a Gaussian random variable, the chance that the real value of $\mathbb{E}[\Delta z]$ differs from μ by at least y satisfies

$$\text{Prob}(|\mathbb{E}[\Delta z] - \mu| \geq y) = 1 - \text{erf}\left(\frac{y}{\sigma_{\rm std}\sqrt{2/M}}\right), \tag{S10}$$

where erf is the error function. For example, when $y = 2\sigma_{\rm std}/\sqrt{M}$, this probability is close to 4.5%.

Note that this procedure requires the precise knowledge of $\sigma_{\rm std}$. When the only source of information is a set of data points, finding $\sigma_{\rm std}$ is a non-trivial task. First, the square root of the sample variance is known to yield a biased estimate (an underestimate) of the true standard deviation. Second, the uncertainty of the variance depends on the variance of the variance, a quantity that scales, when M is large, like $(\mu_4 - \sigma^4_{\rm std})/M$ where μ_4 is the fourth moment. Moreover, as we have seen throughout this paper, for the systems considered here the variance for a given value of the

population size n_t depends on n_t, so different points in an empirical time series are usually picked from distributions with different variances.

These considerations – namely, the importance of accurately knowing the dwell time δ of the environment and the true variance σ^2_{std} of the distribution – hold even if one wishes to estimate only $\mathbb{E}[r]$ from an empirical time series (such as in the various examples discussed in [5]).

The only extra step required for the use of our formulae is the partitioning of the variance between V_{e} and V_{d}/n. To estimate the number of data points required for this, let us consider the simplest case in which the total variance has been determined for two values of n: for $n = n_a$ the variance is V_a, and for $n = n_b$ the variance is V_b. Since

$$V_a = V_{\mathrm{e}} + V_{\mathrm{d}}/n_a, \qquad V_b = V_{\mathrm{e}} + V_{\mathrm{d}}/n_b, \tag{S11}$$

we get

$$V_{\mathrm{e}} = \frac{n_a V_a - n_b V_b}{n_a - n_b}, \tag{S12}$$

and

$$V_{\mathrm{d}} = \frac{n_a n_b (V_b - V_a)}{n_a - n_b}. \tag{S13}$$

Again, the number of points required for a reliable estimate of V_a and V_b is governed by $(\mu_4 - \sigma^4_{\mathrm{std}})/M$, and the error propagates to the estimation of V_{e} and V_{d} according to Eqs. (S12) and (S13) or equivalent formulae.

S4. DETAILS OF THE EXAMPLE MODELS AND OF THE NUMERICAL TECHNIQUES USED

S4.1. Lottery model: discrete-time version

The lottery model of Chesson and Warner [6, 7] is a simple and generic model of ecological dynamics. Its original, discrete-time version generalizes the classical Wright-Fisher model by allowing generations to overlap. Many other ecological models (e.g. Beverton-Holt) can be seen as modifications of the lottery model that account for more realistic processes (age structure, different dynamics for adults and seeds, etc.) but share the same basic structure.

In coexistence theory the lottery model has a particular importance as it exhibits the storage effect. In contrast to the naive viewpoint according to which increased environmental and abundance fluctuations decrease the coexistence time and cause more extinction events, stochasticity may facilitate coexistence due to the negative covariance between environment and competition.

The original formulation of the lottery model neglects demographic stochasticity. In models without demographic stochasticity, a population that starts from a positive abundance can never reach an abundance of strictly zero. This leads to an artificial necessity of having to introduce an arbitrary threshold to define extinction [7]. Here (as in [8]) we extend the lottery model to explicitly include demographic stochasticity, thus making the model more faithful to real-world situations.

The dynamics take place as follows. At the beginning of each timestep t, the population of the invading species is n, where the size of the community is N. Each individual produces a large number of seeds (or larvae, etc.), and the fitness of a species is related to the mean number of seeds produced by an individual. Each invader species individual produces W_i seeds, whereas the mean number of seeds produced by a resident species individual is W_r. The fitness ratio is thus $W_i/W_r \equiv \exp(s)$. If s is positive, the invading species is superior, when s is negative it is inferior. s depends on time. To simulate it, we picked, at each step, a number s_t from a normal distribution with mean s_0 and variance σ^2.

After the seed-production step, any individual in the community dies with a probability δ, leaving on average $N\delta$ empty gaps to be recruited. Seed dispersal is practically infinite, so spatial effects play no role (i.e., the dynamics are well-mixed). Therefore, the chance P_t^{win} of the invader species to recruit any given gap is dictated by its share in the seed bank, namely,

$$P_t^{\mathrm{win}} = \frac{n_t e^{s_t}}{n_t e^{s_t} + N - n_t} \approx \frac{n_t}{N} e^{s_t}. \tag{S14}$$

Accordingly, in each step the mean number of invaders satisfies $n_{t+\tau} = n_t(1-\delta) + P_t^{\mathrm{win}} N\delta$.

A significant point to note here is that for the discrete-time lottery model, the length of a single time step is

τ generations, because τ is taken to be the dwell time of the environment (in units of generation time), and the environment is taken to change per time step in an uncorrelated manner. This implies that in this model the dwell time τ must equal δ, the probability of an individual to die in a time step. This is because if an individual dies in a time step with a probability δ, then on average a fraction δ of the entire population dies per time step, which means that the entire population dies on average every $1/\delta$ time steps. A generation is defined as the time in which the entire population is replaced by new individuals, so $1/\delta$ time steps equal one generation, which means that one time step equals δ generations. Therefore, in this model, $\tau = \delta$.

Demographic stochasticity in this system has two sources. First, the number of invader species individuals that die in each step is not precisely $n_t\delta$, but is picked from a binomial distribution whose mean is $n_t\delta$. Analogously, the number of resident species deaths is distributed binomially around $(N - n_t)\delta$. Second, for a given number of gaps G (arising from the death of some invader and some resident species individuals) the number recruited by the invader species is again distributed binomially around $P_t^{\text{win}}G$.

S4.2. Lottery model: continuous-time (Moran) version

In the discrete-time version of the lottery model, as described above, δ plays two roles: first, it dictates the intrinsic dynamics of the population that take place through consecutive events of death and recruitment, each involving (on average) δN individuals. Second, δ equals τ, the dwell time of the environment. This double function of δ leads to a connection between the intrinsic dynamics of the population and the dynamics of the environment. To lift this restriction one may consider a continuous-time version of the lottery model [9, 10].

In this version, in each elementary step, only one individual is chosen at random to die. The resulting gap is recruited by an offspring of the focal species with probability P_t^{win} [Eq. (S14)] and by an offspring of the resident species with probability $1 - P_t^{\text{win}}$. In each elementary step, the environment switches with probability $1/(N\tau)$, so the persistence time is picked from a geometric distribution whose mean is the dwell time τ.

S4.3. Forest dynamics model

The forest dynamics model, as employed by [11, 12], is based on Leslie-Gower dynamics. Growth is divided into a sapling stage and an adult stage. The sapling dynamics are described by

$$s_i(t+1) = \frac{f_i s_i(t)}{1 + \beta_{ii} s_i(t) + k_{ii} x_i(t)} + \frac{R_i(t) x_i(t)}{1 + \sum\limits_{j=1}^{S} \alpha_{ij}(t) x_j(t) R_j(t)}. \tag{S15}$$

Here the subscripts (i or j) label the species and t denotes the time, measured in years. The parameter s stands for the sapling density, f represents the sapling fraction surviving from a year to the following one, R denotes the rate at which seeds (or seedlings) are generated, and x denotes the frequency of the adults. S is the total number of species in the system, and β, k and α quantify the competition faced by the saplings (of a given species) from other saplings of their own species and from the adults and the saplings of the other species.

The two terms on the right hand side in Eq. (S15) correspond to the saplings surviving from previous years, and to new saplings that are generated by the adults. In order to focus on the effect of environmental variability, we have taken the values of the competition parameters to be $\alpha_{ij} = 1$ and $\beta_{ii} = k_{ii} = 0$ for all i, j in our simulations, mimicking the approach employed by [11, 12].

The adults in the model follow dynamics resembling the lottery model [6],

$$x_i(t+1) = dx_i(t) + (1-d)\frac{s_i(t)}{\sum\limits_{j=1}^{S} s_j(t)}. \tag{S16}$$

Here d denotes the fraction of adults of a given species that survives in each timestep. The ratio of the frequency of the saplings of a species to the total number of saplings across all species determines the probability of that species to take up an open adult gap. In the limit $f_i = 0$ for all i, the model loses its "memory" of the saplings surviving from previous years and converges to the lottery model. It is due to this long-term memory that in the forest dynamics model the dwell time τ of the environment is not directly related to the chance of an individual to die during τ, as in the two versions of the lottery model.

S4.4. Numerical simulations

We ran Monte Carlo simulations of the three models described above in order to check the predictions of our invasion formula.

In the discrete-time lottery model, the number of dying individuals from the invading species, which had an abundance n_t at the beginning of a timestep, was picked randomly from a binomial distribution with n_t trials and the chance of success δ. Similarly, the number of individuals dying from the resident species was picked from a binomial distribution with $N - n_t$ trials and the chance of success δ. This meant that the average total number of deaths (across both the species) in each timestep was $N\delta$, as desired. If $d_{1,t}$ and $d_{2,t}$ denote the number of deaths thus suffered by the invading and resident species, then the number of births for the invading species was picked randomly from a binomial distribution with $d_{1,t} + d_{2,t}$ trials and the chance of success P_t^{win}. The resident species was allowed to fill up all the remaining empty gaps, thus preserving the total population as N individuals after every timestep.

For the Leslie-Gower model, a detailed exposition of our simulation technique (including Matlab codes) and of the parameters used in these simulations is provided in Supplement S8 below.

S5. COMPARISON BETWEEN SIMULATION RESULTS AND THE TWO FORMULAE

In this section we present many plots for the lottery model, comparing the results from Monte Carlo simulations (as explained in Supplement S4), from our WKB-based formula from Eq. (20) in the main text, and from our diffusion approximation (DA)-based formula from Eq. (6) in the main text. In the following plots, the simulations are marked in blue circles connected by full lines, the WKB-based formula results in yellow diamonds connected by dshed lines, and the DA-based formula results in red circles connected by dashed dotted lines. In each plot the value of the mean selection parameter s_0 and of δ (the probability of an individual to die per time step, which equals the dwell time τ in the lottery model) is kept fixed, while σ (the log-amplitue of environmental variations) varies on the horizontal axis (see Supplement S4 for details on the meanings of the different parameters). There are sixteen plots shown, corresponding to all combinations of $s_0 \in \{-0.2, 0, 0.2, 0.4\}$ and $\delta \in \{0.1, 0.3, 0.5, 0.7\}$ (the values for each plot marked at the top of the plot).

The general takeaway from these plots is that the DA-based formula works only for small levels of environmental variations, and only for weak selection. The WKB-formula works well for a much wider range of parameters.

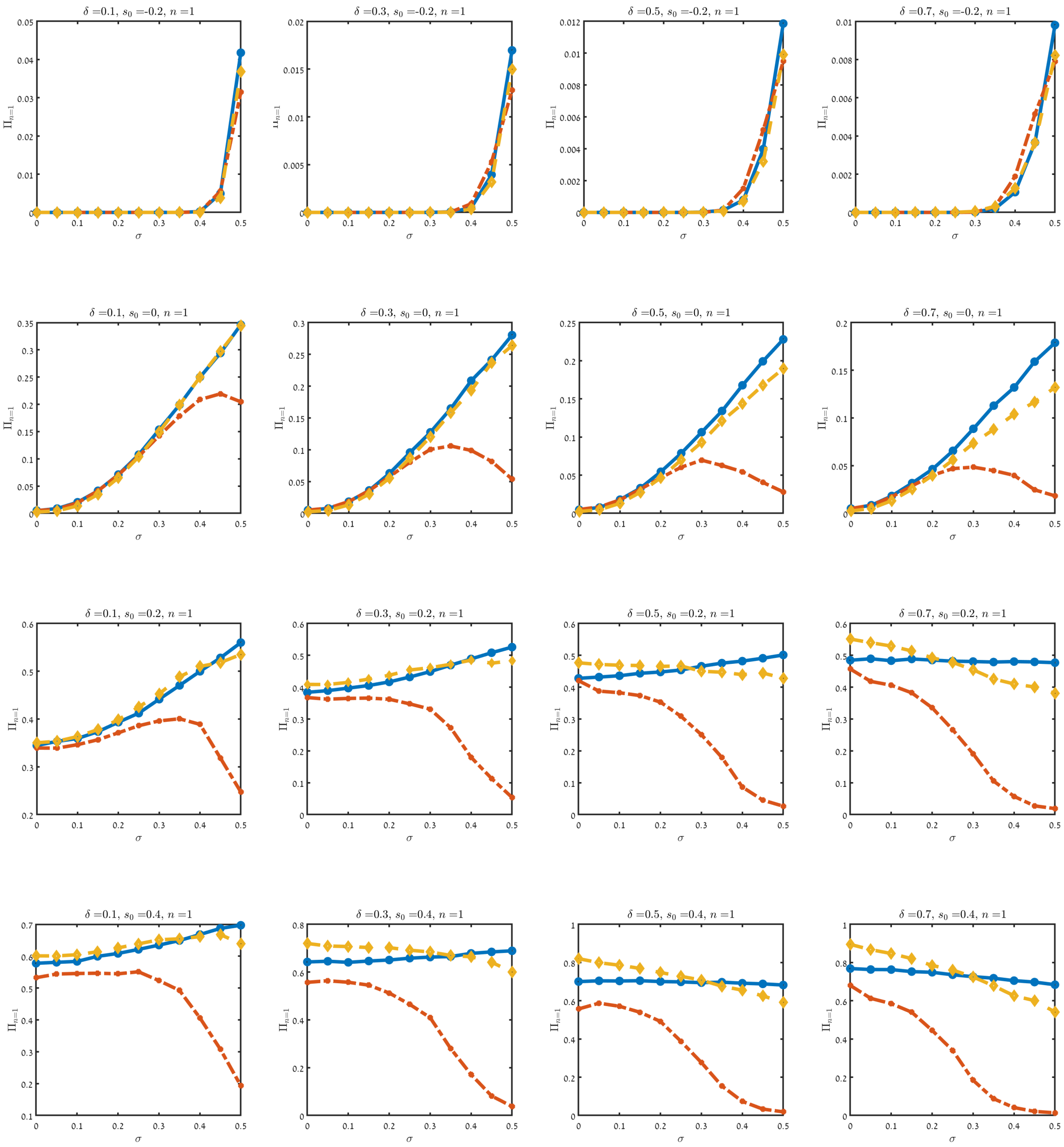
δ =0.1, s0 =-0.2, n =1
δ =0.3, s0 =-0.2, n =1
δ =0.5, s0 =-0.2, n =1
δ =0.7, s0 =-0.2, n =1
δ =0.1, s0 =0, n =1
δ =0.3, s0 =0, n =1
δ =0.5, s0 =0, n =1
δ =0.7, s0 =0, n =1
δ =0.1, s0 =0.2, n =1
δ =0.3, s0 =0.2, n =1
δ =0.5, s0 =0.2, n =1
δ =0.7, s0 =0.2, n =1
δ =0.1, s0 =0.4, n =1
δ =0.3, s0 =0.4, n =1
δ =0.5, s0 =0.4, n =1
δ =0.7, s0 =0.4, n =1
Πn=1
σ

S6. SUPPLEMENTS FOR FIG. 3 OF THE MAIN TEXT

In Fig. 3 of the main text we presented the chance of invasion $\Pi_{1\to200}$, as obtained from numerical solutions of the discrete-time lottery model, for different values of the dwell time τ (which equals δ, the probability of an individual to die per time step) and the amplitude of fitness variations σ. We plotted $\Pi_{1\to200}$ against the invasion parameter $\mathbb{E}[r]$ and obtained scattered results, without a specific trend associated with the mean growth rate. When we plotted $\Pi_{1\to200}$ against the predictions of Eq. (20), we obtained a nearly perfect data collapse. In Fig. 3 we used $n_f = 200$. Here we present corresponding figures for larger and smaller values of n_f, to indicate how it may affect the results.

For $n_f = 500$, the data-collapse is comparable to the results obtained for $n_f = 200$, as seen in Fig. S1.

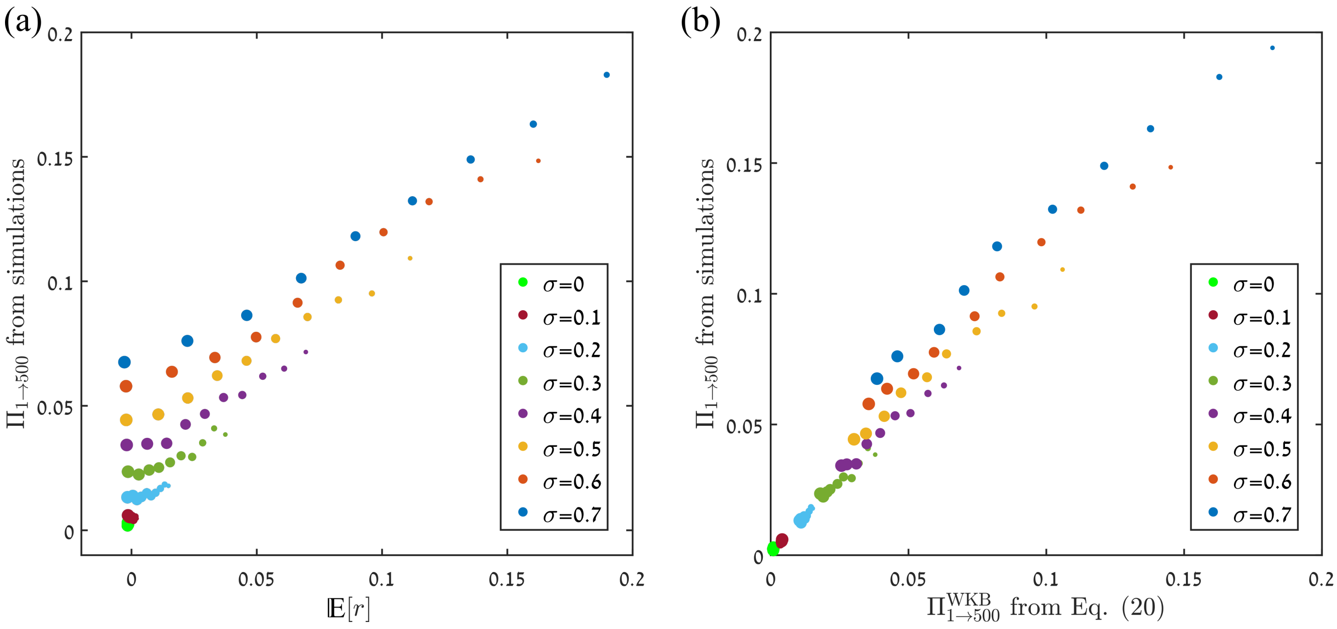

FIG. S1. The chance of invasion for $n_f = 500$. All other parameters, as well as the size code for τ, are identical to those used in Fig. 3 of the main text.

When $n_f = 20$ (Fig. S2) clear deviations appear in the predictions of the WKB-based formula, although they are still correct in the order of magnitude compred to the true chance of invasion. The reason for this relative poor performance of the WKB-based formula is that when n_f is very small, it is harder to obtain the parameters E_0, V_d and V_e from abundance time series. In such a case, it is advisable to use the formula from Eq. (6) in the main text instead, which is based on the diffusion approximation. While this formula is limited by the requirements of the diffusion approximation of being valid only for small selection and stochasticity parameters, the determination of the parameters required for its use is not susceptible to the same errors as the WKB approach. That is, the procedure for the calculation of $\mathcal{E}$, $\mathcal{V}_d$ and $\mathcal{V}_e$ is robust even for small values of n_f. For this reason, the formula in Eq. (6) compares quite well to the true chance of invasion, as seen in Fig. S2(c), as long as σ, the log-amplitude of the environmental

variations, is not large.

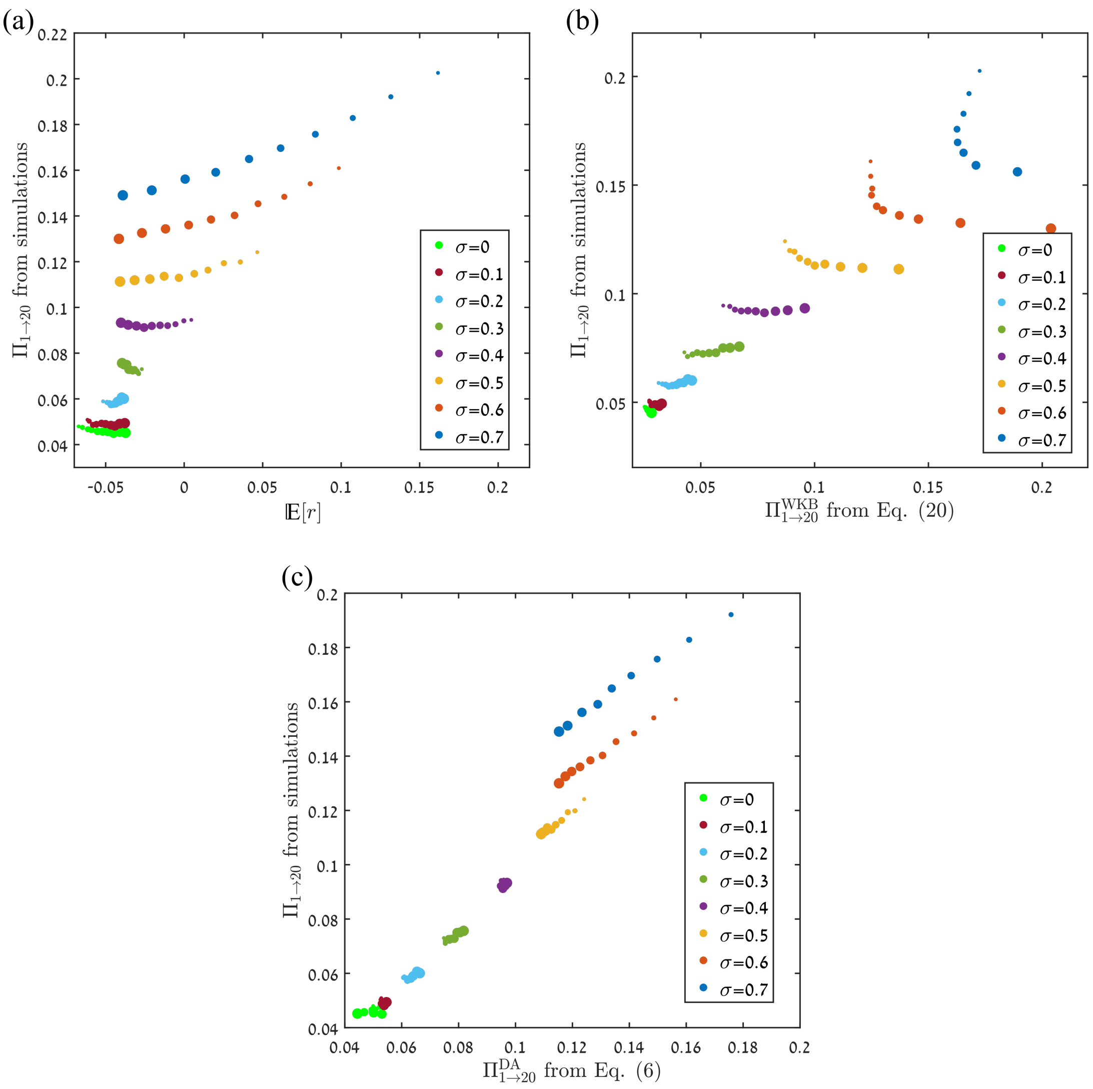

FIG. S2. The chance of invasion for $n_{\mathrm{f}} = 20$. All other parameters, as well as the size code for τ, are identical to those used in Fig. 3 of the main text. Panels (a) and (b) are similar to those in Fig. 3 of the main text, but here there is an additional panel, panel (c), which compares the true chance of invasion with the prediction of Eq. (6) in the main text, which is based on the diffusion approximation. Since n_{f} is so small, the DA-based formula performs noticeably better than the WKB-based formula – i.e., the values for the chance of invasion it predicts match closely the true chance of invasion on the vertical axis – as long as σ is not too large. The reason why the WKB-based formula performs less well than in Fig. 3 of the main text and in Fig. S1 here, is that for this small value of n_{f} it is more prone to errors in the calculation of the parameters E_0, V_{d} and V_{e}.

Figs. S1 and S2 also indicate that as n_{f} decreases, the parameter τ diminishes in importance. This is so because in the extreme case, as n_{f} becomes very small, there is no dependence of the chance of invasion on τ: a population reaches the invasion point, or goes extinct, before the environment flips. As explained in the main text, this corresponds to the "quenched" regime of Mustonen and Lässig [13], which is not covered by our theory. Even so, Fig. S2 suggests that the disagreement is not large even for n_{f} as small as 20.

S7. SUPPLEMENTS FOR FIG. 4 OF THE MAIN TEXT

In panel (a) of Fig. 4 of the main text we showed the chance of invasion in the Moran model as a function of the starting population n for different values of the fitness advantage s_0, where $\sigma = 0.3$, $\tau = 0.1$, $N = 5000$ and $n_{\rm f} = 200$. Here we show the same figures for two other values of N.

As N increases, the agreement of the theory with the simulations grows likewise, because the ratio $n_{\rm f}/N$ is smaller, so density-dependent effects in the invasion regime are even weaker. This is seen in the (slightly) better agreement of the simulation results and the curves resulting from our WKB-based formula for $N = 10000$, shown in Fig. S3.

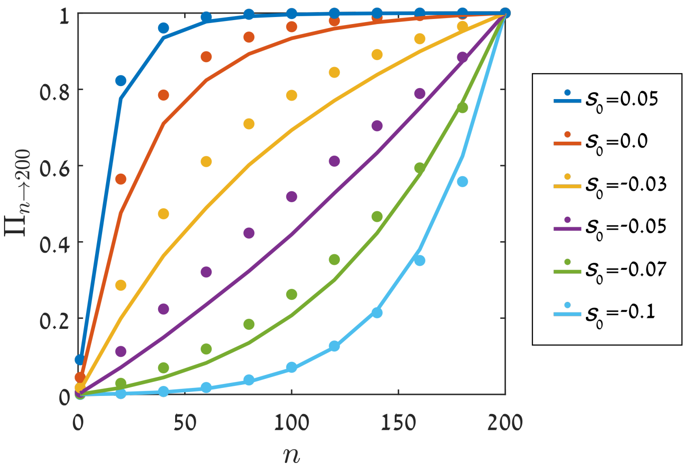

FIG. S3. The chance of invasion as a function of the initial abundance n, with $N = 10000$ and $n_{\rm f} = 200$. The Monte Carlo simulations are marked as circles and the theoretical predictions (Eq. (20) of the main text) as full lines. All other parameters are identical to those used in Fig. 4(a) of the main text.

On the other end, as N decreases (and $n_{\rm f}$ is held constant), density-dependent effects become more prominent and the agreement between our formula and the numerical results gets weaker. For example, for $N = 1000$ (Fig. S4), the deviations are a little more pronounced than those observed in Fig. 4 of the main text.

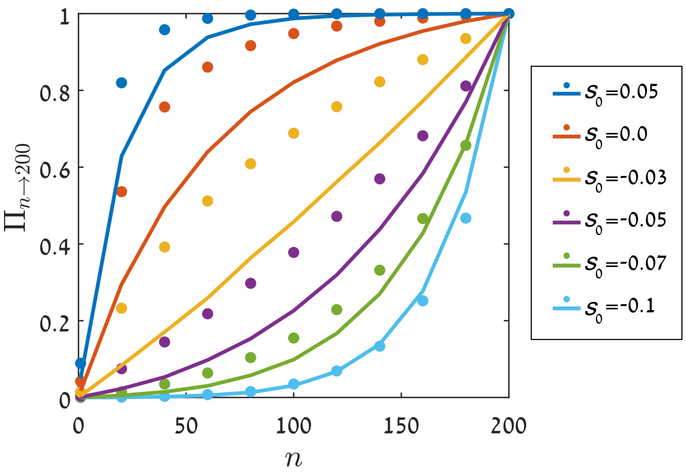

FIG. S4. The chance of invasion as a function of the initial abundance n, with $N = 1000$ and $n_{\mathrm{f}} = 200$. The Monte Carlo simulations are marked as circles and the theoretical predictions (Eq. (20) of the main text) as full lines. All other parameters are identical to those used in Fig. 4(a) of the main text.

S8. SUPPLEMENTS FOR FIG. 5 OF THE MAIN TEXT

To demonstrate the effectiveness of our invasion formula, and the procedure involved in using it, we considered in Fig. 5 of the main text a generic model of forest dynamics, namely the Leslie-Gower model, as employed in [11, 12], and the accompanying empirical time series for the recruitment rates of the two species *Spondias mombin* and *Spondias radlkoferi* as presented in [11]. Under the original parameters of [11] an invasion of one of the species is nearly certain, and occurs very fast, even for very small values of n. Therefore, in our demonstration we modified some of the parameters (namely, the survival probability of adults and of saplings, and the overall strength of recruitment fluctuations) while preserving the proportions and the cross-correlations of the original recruitment time series. Here we explain the procedure in detail and provide an annotated MATLAB code for each step.

Our starting points are equations (S15) and (S16). When the model is limited to two species (indexed by i) and the simplifications made in [11], as detailed in Supplement S4, are adopted, the deterministic map for sapling dynamics is given by

$$s_i(t+1) = f s_i(t) + \frac{R_i(t)x_i(t)}{1 + x_1(t)R_1(t) + x_2(t)R_2(t)}, \tag{S17}$$

where $s_i(t)$ is the density of saplings of the species i at time t, f is the fraction of surviving saplings (of each species) from one year to the next, and $R_i(t)$ is the recruitment rate of species i at time t. Similarly, the deterministic map for adult dynamics is

$$x_i(t+1) = d x_i(t) + (1-d)\frac{s_i(t)}{s_1(t) + s_2(t)}, \tag{S18}$$

where $x_i(t)$ is the density of the adults of species i at time t, and d is the fraction of surviving adults (of each species) from one year to the next.

To obtain Fig. 5 of the main text, we first performed Monte Carlo simulations of the above model, calculating from them the chance of a population of n individuals to grow in abundance to n_{f} before going extinct. In our simulations only the density of adults was quantized, i.e., demographic stochasticity entered the model only at the level of the adult dynamics. The sapling density was taken as a continuous variable, since the number of saplings is typically much larger than the number of adult trees. In section S8.1 we provide the MATLAB code for this MC simulation. A typical outcome is presented in Fig. S5.

In order to use Eq. (20) of the main text one must find τ, E_0, V_{e} and V_{d}. To do that we generated a long time series of the same dynamics as described in equations (S17) and (S18), using the code provided in section S8.2 below. We stipulated reflecting boundary conditions, so that $n = 0$ became $n = 1$ and $n = N$ became $n = N - 1$. This was done to make it easier to obtain adequately long time series. Typical time series obtained are shown in Fig. S6.

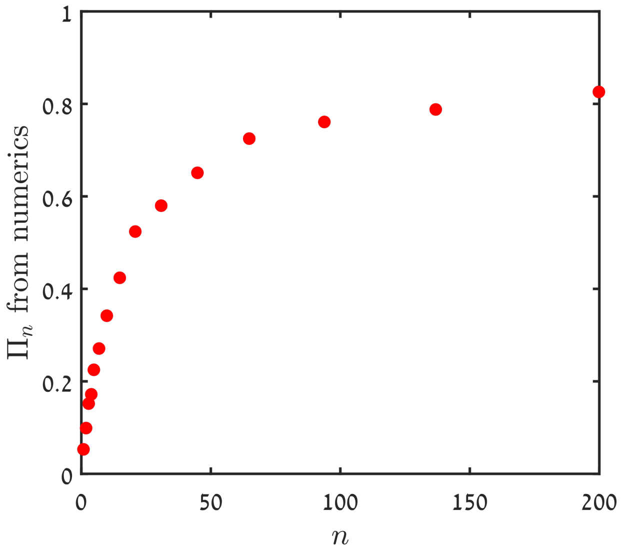

FIG. S5. The chance of invasion ($n_\mathrm{f} = 1000$) for species 1 vs. its initial abundance n, averaged over 1000 trials of Monte Carlo simulations.

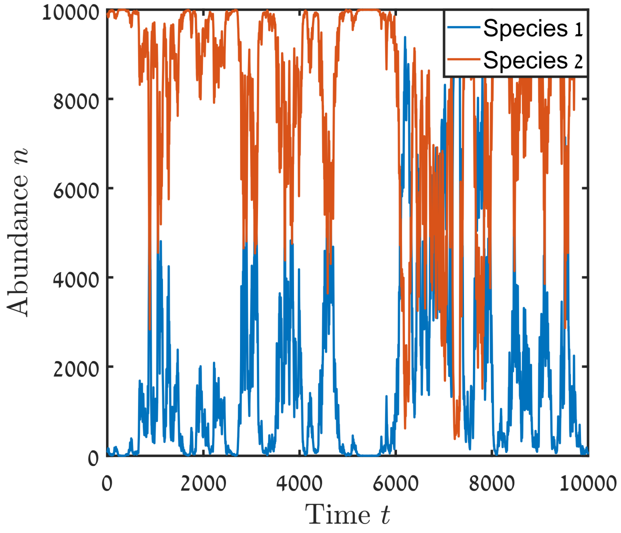

FIG. S6. A time series for the abundances of the two species in the forest dynamics model, obtained from the Matlab code presented in section S8.2, between $t = 0$ and $t = 10000$.

These time series were then translated to the logit parameter $z = \log n_t/(N - n_t)$, in order to find the autocorrelation time of the time series (and, thereby, the dwell time). As explained in [1], the logit parameter is the most suitable for measuring abundance variations at all densities n_t/N. Note that in all our discussion in the main text and in Supplements S2 and S9 we have considered only the invasion regime $n_t \ll N$, where $z \approx \ln n_t$ (ignoring the constant term $-\ln N$, since it disappears in Δz), but to calculate the autocorrelation time using general time series, the logit variable is to be preferred.

We then plotted the autocorrelation function for the time series of each species (Fig. S7). Assuming that this function decays like $\exp(-t/\xi)$, we identified ξ from a linear regression in a log-linear plot. This step requires some

care, since the exponential behavior is sometimes limited to a narrow range of times, but a visual identification of this regime is usually easy. For a given ξ, $\tau = 2\xi$ is the dwell time.

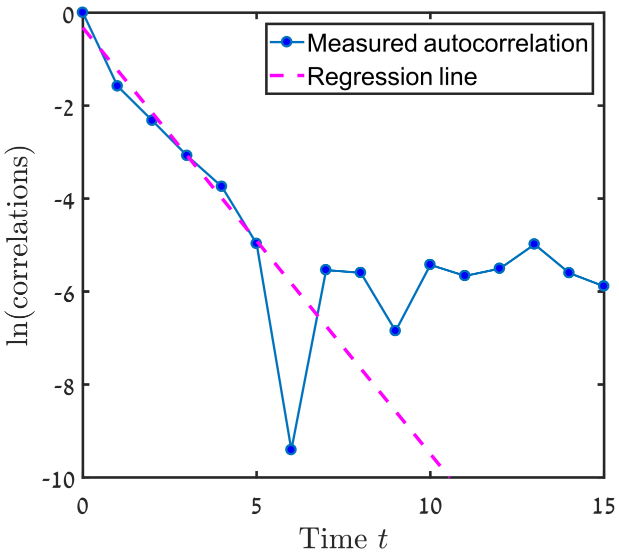

FIG. S7. The calculated autocorrelation function is presented on a log-linear plot. Linear regression of the first six points (dashed line) here yields a negative slope of about -0.9, suggesting a dwell time between 2 and 3. We have rounded the dwell time to the higher integer, as the measured autocorrelations in the abundance variations are expected to decay slightly faster than the correlations associated with the actual environmental variations because of the effect of demographic stochasticity.

Once the dwell time τ was identified, we calculated $\mathbb{E}[\Delta z]$ and the variance Var[Δz] for each n, and plotted $\mathbb{E}[\Delta z]$ vs. n (Fig. S8) and $n \times$ Var[Δz] vs. n (Fig. S9). The code for this is presented in section S8.3. Fitting these plots with straight lines, as explained in the captions of the two figures, we obtained E_0, V_e and V_d. Plugging the values of these parameters into Eq. (20) of the main text we obtained the datasets used to generate Fig. 5 of the main text.

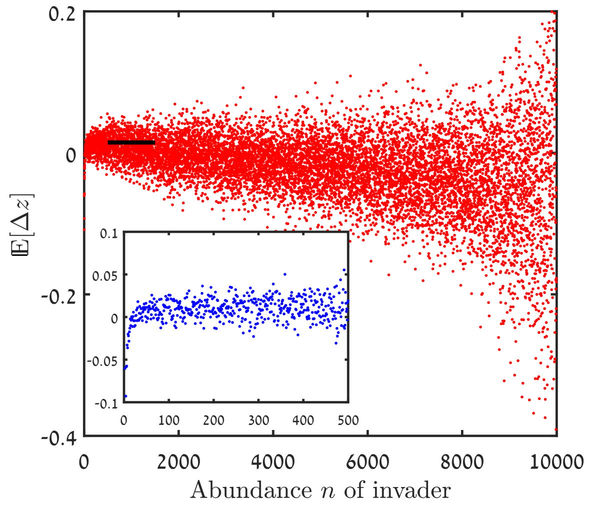

FIG. S8. The main panel shows $\mathbb{E}[\Delta z]$ vs. n. When the small-n region of the plot is magnified (inset), a sharp increase in $\mathbb{E}[\Delta z]$ with n is observed for small n, due to the effect of demographic stochasticity as explained in the main text. Avoiding these small values of n, we applied linear regression to $\mathbb{E}[\Delta z]$ in the small n/N regime and used the intercept (thick black line) as the measured value of E_0.

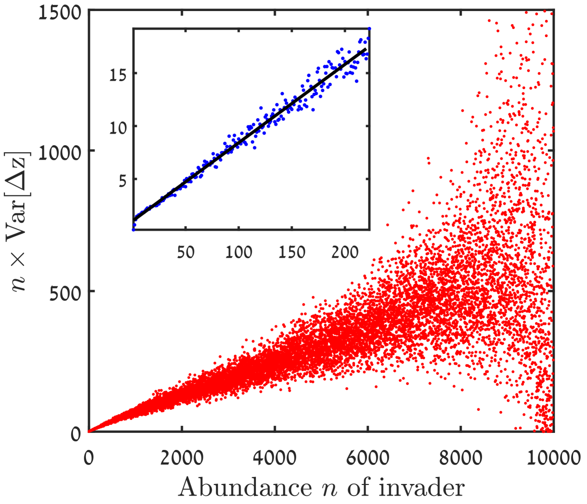

FIG. S9. $n \times \mathrm{Var}[\Delta z]$ vs. n. A linear regression fit at small n values (inset, regression line shown in black) yields V_e as its slope and V_d as its intercept.

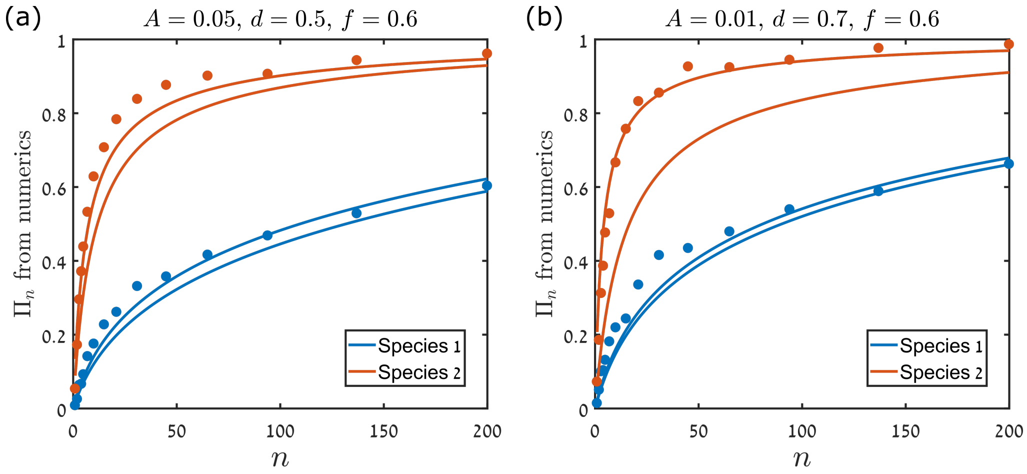

FIG. S10. The chance of invasion for the forest dynamics model as measured in numerical simulations (circles) and as predicted by Eq. (20) of the main text (lines). In the title of each subfigure, A is an overall constant that multiplies the recruitment rate time series of each species, and d and f are as defined above. For each set of parameters, different lines correspond to different estimations of the parameters E_0, V_e and V_d, as obtained from two successive applications of the procedure described above (using the code presented in the sections S8.2 and S8.3). In panel (a), these estimations yield, for species 1, $E_0 = -0.023278$, $V_e = 0.29463$ and $V_d = 2.281$ in one attempt, and $E_0 = -0.015824$, $V_e = 0.2985$ and $V_d = 2.118$ in the second attempt. For species 2, we obtained $E_0 = 0.09552$, $V_e = 0.2815$ and $V_d = 1.9924$ in the first attempt and $E_0 = 0.10592$, $V_e = 0.31971$ and $V_d = 1.3593$ in the second attempt. Similarly, in panel (b), for species 1, the first measurement attempt yielded the parameters $E_0 = 0.00058729$, $V_e = 0.13869$ and $V_d = 1.4524$, while the second attempt yielded $E_0 = 0.0026402$, $V_e = 0.14099$ and $V_d = 1.4093$. For species 2, we found $E_0 = 0.050777$, $V_e = 0.15023$ and $V_d = 1.5319$ in the first attempt and $E_0 = 0.059525$, $V_e = 0.16207$ and $V_d = 0.44297$ in the second attempt.

There is some subjectivity involved in the whole process of finding the parameters needed for Eq. (20) from abundance time series, like in the identification of the exponential part of the autocorrelation function or of the regime of n-values where n/N is small enough to be in the invasion regime but not so small that the initial part where $\mathbb{E}[\Delta z]$ sharply increases with n is included. As demonstrated in Fig. S10, this subjectivity does not affect the final

results too much, and in all the cases we have checked, the final invasibility value has not varied by more than about 20% (and usually much less than that) owing to the differences in measuring the Eq. (20) parameters. Ideally, one should take the mean values of the measured parameters over a few different instances of following the procedure, in order to minimise errors in them.

In Fig. 5 of the main text we plotted the results for $A = 0.01$ (where A is an overall constant that multiplies the recruitment rate time series of each species) and for various values of f, n and d against $\mathbb{E}[r]$ and against our formula (Eq. (20) of the main text). Here (Fig. S11) the same figures are presented again. The E_0, V_e and V_d parameters in both the panels were calculated exactly once for each dataset, with E_0 obtained by fitting the regime $n \in [800..2200]$ and with V_e and V_d found by fitting the regime $n \in [0..100]$. While the resulting agreement obtained between the simulation results and our formula, in Fig. S11(b) (and in Fig. 5(b) of the main text) is not perfect, the deviations are not large, and the predictive ability of our formula is clearly significantly better than that of E_0 (Fig. S11(a), and Fig. 5(a) of the main text).

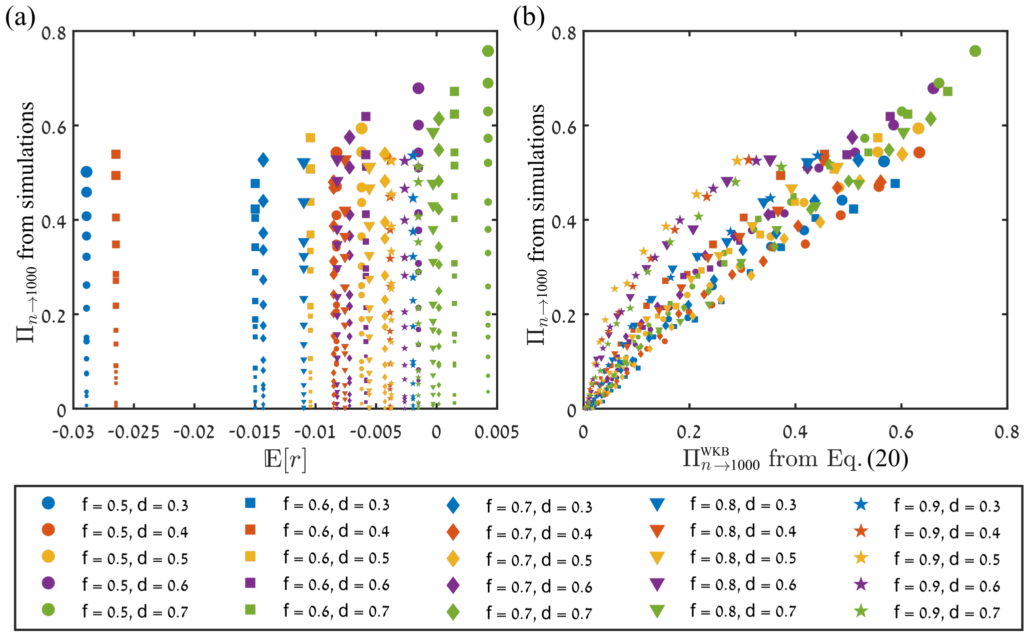

FIG. S11. Same as Fig. 5 of the main text.

S8.1. Matlab code for the chance of invasion: Monte Carlo simulations

Here we provide the MATLAB code to find the chance of invasion of a species in the Leslie-Gower model, as explained in Supplement S4. Explanatory comments in the code are shown in green.

```
% The values of the recruitment rates R(t), for the two species Spondias
% mombin and Spondias radlkoferi , as provided in Usinowicz et al. (Ecology,
% 2012).
R10 = [0.1761 0.1558 0.6239 0.2779 0.1761 0.2576 1.1385 0.2769 ...
    1.3846 1.0154 1.6308 1.2923 2.9846 3.5434];
R20 = [0.3503    2.6154    1.2615    1.4154    0.6154    1.2000  ...
    0.3385 0.3077    2.1538    0.5846    0.7385    1.7846  ...
    0.9538    0.4000];

% As in Usinowicz et al. (Ecology, 2012) & Usinowicz et al. (Nature, 2017),
% these values are normalized such that the mean recruitment rates of the
% two species are equal and only their temporal fluctuations and the
% correlations between them are important. We multiply the recruitment
% rates by a constant factor A to be able to modify the overall strength
% of these fitness variations.
% focspec is the identity of the focal (i.e., invading) species (1 or 2).
focspec = 1;
A = 0.05;
if focspec == 1
    R1 = A*(R10/mean(R10));
    R2 = A*(R20/mean(R20));
else
    R1 = A*(R20/mean(R20));
    R2 = A*(R10/mean(R10));
end

% Basic parameters of the model.
mem = length(R1);
N = 10000;
f = 0.6;
d = 0.5;
% nf is the target abundance above which invasion is declared.
nf = 1000;
% exper is the number of trials for each initial n.
exper = 1000;
% nb is the vector of initial n values.
nb = ceil(exp(linspace(0,log(200),15)));
% win counts cases in which the population reaches nf before extinction.
win = zeros(1,length(nb));

for k1 = 1:length(nb)
    n0 = nb(k1)
    for k=1:exper
        n1 = n0;
        n2 = N-n1;
        s1 = n1/N;
        s2 = n2/N;
        while n1>0 && n1<nf
            x1 = n1/N;
            x2 = n2/N;
            % Pick a weather for a given year.
```

```
            year = ceil(mem*rand);
            % Sapling dynamics (no discretization)
            s1 = f*s1+R1(year)*x1/(1+x1*R1(year)+x2*R2(year));
            s2 = f*s2+R2(year)*x2/(1+x1*R1(year)+x2*R2(year));
            sr = s1/(s1+s2);
            % The function bnldev(M,p), picks a binomial deviate from
            % M attempts with the success rate p. Any other generator of
            % binomial deviates may be used instead.
            death1 = bnldev(n1,1-d);
            death2 = bnldev(n2,1-d);
            tot_death = death1+death2;
            birth1 = bnldev(tot_death,sr);
            n1 = n1-death1+birth1;
            n2 = n2-death2+(tot_death-birth1);
        end
        % Record successful invasions.
        if n1>nf-1
            win(k1) = win(k1)+1/exper;
        end
    end
end
figure;plot(nb,win,'o')
hold all
```

A typical outcome of the above code is shown in Fig. S5, and is repeated here for ease of access.

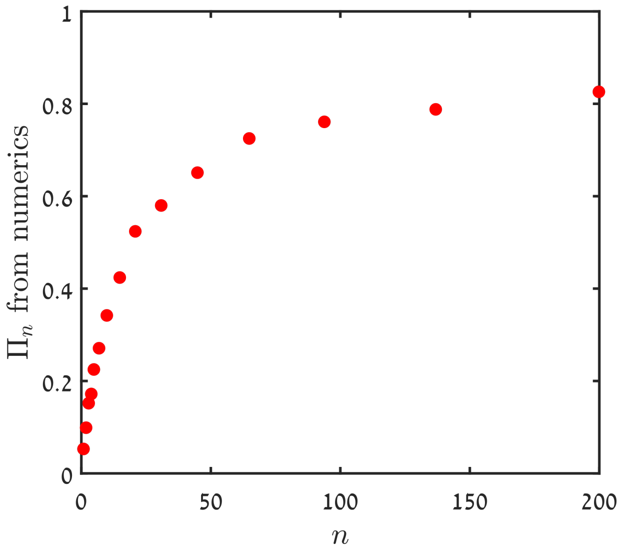

FIG. S12. The chance of invasion ($n_\mathrm{f} = 1000$) for species 1 vs. its initial abundance n, averaged over 1000 trials of Monte Carlo simulations.

S8.2. Matlab code to generate long time series and calculate dwell times

The following MATLAB code generates long abundance time series and calculates the autocorrelation function. Explanatory comments are shown in green.

```
% The values of the recruitment rates R(t), for the two species Spondias
% mombin and Spondias radlkoferi, as provided in Usinowicz et al. (Ecology,
% 2012).
R10 = [0.1761 0.1558 0.6239 0.2779 0.1761 0.2576 1.1385 0.2769 ...
    1.3846 1.0154 1.6308 1.2923 2.9846 3.5434];
R20 = [0.3503     2.6154     1.2615     1.4154     0.6154     1.2000 ...
    0.3385 0.3077     2.1538     0.5846     0.7385     1.7846 ...
    0.9538     0.4000];

% As in Usinowicz et al. (Ecology, 2012) & Usinowicz et al. (Nature, 2017),
% these values are normalized such that the mean recruitment rates of the
% two species are equal and only their temporal fluctuations and the
% correlations between them are important. We multiply the recruitment
% rates by a constant factor A to be able to modify the overall strength
% of these fitness variations.
% focspec is the identity of the focal (i.e., invading) species (1 or 2).
focspec = 1;
A = 0.05;
if focspec == 1
    R1 = A*(R10/mean(R10));
    R2 = A*(R20/mean(R20));
else
    R1 = A*(R20/mean(R20));
    R2 = A*(R10/mean(R10));
end

% Basic parameters of the model.
mem = length(R1);
N = 10000;
f = 0.6;
d = 0.5;
% TT is the length of the timeseries, while spp1 and spp2 record the
% species abundances.
TT = 1000000;
spp1 = zeros(1,TT);
spp2 = zeros(1,TT);
% Initial conditions
n1 = 100;
n2 = N-n1;
s1 = n1/N;
s2 = n2/N;

for j=1:TT
    x1 = n1/N;
    x2 = n2/N;
    year = ceil(mem*rand);
    s1 = f*s1+R1(year)*x1/(1+x1*R1(year)+x2*R2(year));
    s2 = f*s2+R2(year)*x2/(1+x1*R1(year)+x2*R2(year));
    sr = s1/(s1+s2);
    % The function bnldev(M,p), picks a binomial deviate from
    % M attempts with the success rate p. Any other generator of
```

```
    % binomial deviates may be used instead.
    death1 = bnldev(n1,1-d);
    death2 = bnldev(n2,1-d);
    tot_death = death1+death2;
    birth1 = bnldev(tot_death,sr);
    n1 = n1-death1+birth1;
    n2 = n2-death2+(tot_death-birth1);
    % Reflecting boundary conditions
    if n1==0
        n1=1;
        n2 = N-1;
    else if n2==0
            n2=1;
            n1 = N-1;
        end
    end
    spp1(j) = n1;
    spp2(j) = n2;
end
% A given history
figure; plot([1:TT],spp1,[1:TT],spp2)

% Find correlation time using data generated above.
% Measures changes in the logit parameter.
logit = log(spp1(1:end)./(N-spp1(1:end)));
change=logit(2:end)-logit(1:end-1);

nyears = 20;
[c1,c2]=autocorr(change,nyears);
figure;plot(c2,log(c1))
```

An example of abundance time series for the two species, generated by the above code, is presented in Fig. S6 and is repeated here for ease of access.

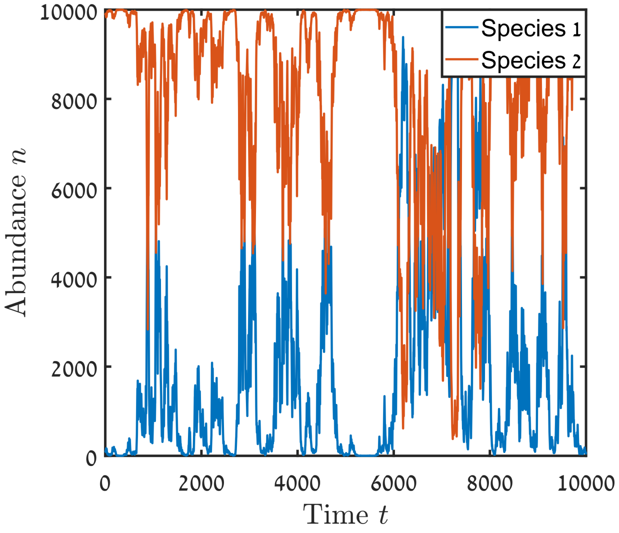

FIG. S13. A time series for the abundances of the two species in the forest dynamics model, between $t = 0$ and $t = 10000$.

The autocorrelation function and its fit are shown in Fig. S7, and are also repeated here.

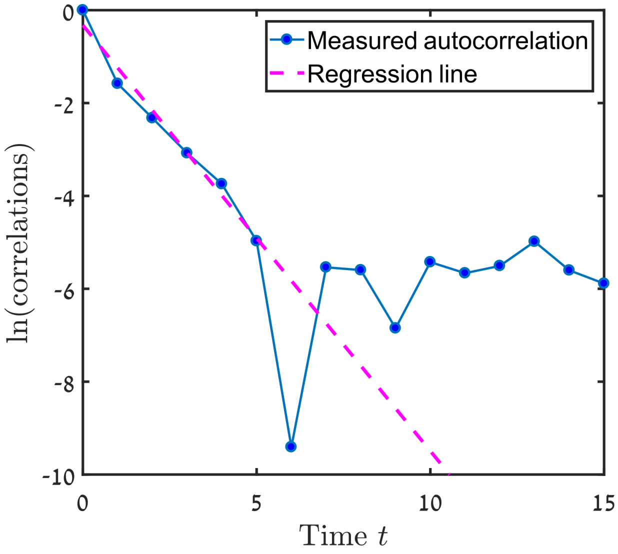

FIG. S14. The calculated autocorrelation function is presented on a log-linear plot. Linear regression of the first six points (dashed line) here yields a negative slope of about -0.9, suggesting a dwell time between 2 and 3. We have rounded the dwell time to the higher integer, as the measured autocorrelations in the abundance variations are expected to decay slightly faster than the correlations associated with the actual environmental variations because of the effect of demographic stochasticity.

S8.3. Matlab code to find $\mathbb{E}[\Delta z]$, V_{e} and V_{d}

The following MATLAB code calculates the mean value E_0 and its variance for each value of n. Again the explanatory comments are shown in green.

```
% The following block adjusts the dwell time (called 'dwell') depending on
% the value of f. This correspondence was found by running the previous
% code (section S7.2) for different f values.
if f==0.5
    dwell = 2;
elseif f==0.6
    dwell = 3;
elseif f==0.7
    dwell=4;
elseif f==0.8
    dwell=5;
elseif f==0.9
    dwell=6;
end

logit = log(spp1(1:end)./(N-spp1(1:end)));
change1=logit(1+dwell:end)-logit(1:end-dwell);

% dz measures the change in Delta z, and dz2 measures the change in its
% squared value.
dz = zeros(1,N);
dz2 = zeros(1,N);
% nvisits keeps an account of the number of times each particular value of
% n is visited.
nvisits = zeros(1,N);
for j=1:length(change1)
    dz(spp1(j)) = dz(spp1(j))+change1(j);
```

```
    dz2(spp1(j)) = dz2(spp1(j))+change1(j)^2;
    nvisits(spp1(j)) = nvisits(spp1(j))+1;
end
% aa keeps a track of those nvalues that are visited at least once.
aa = find(nvisits>1);
% cc finds the variance of Delta z.
cc = (dz2(aa)./nvisits(aa))-(dz(aa)./nvisits(aa)).^2;
figure; plot(aa,dz(aa)./nvisits(aa),'.');
figure; plot(aa,aa.*cc,'.');
```

The outcomes of the code above are plots of E_0 vs. n (Fig. S8) and $n \times \mathrm{Var}[\Delta z]$ vs. n (Fig. S9). For convenience, Figs. S8 and S9 are reproduced here.

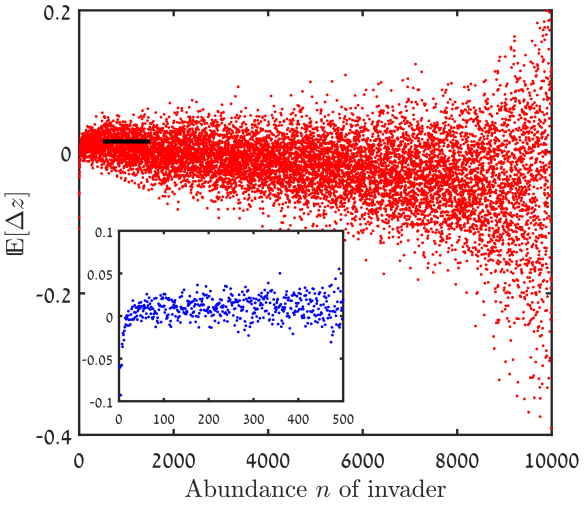

FIG. S15. Main panel: $\mathbb{E}[\Delta z]$ vs. n. When the small-n region of the plot is magnified (inset), a sharp increase in $\mathbb{E}[\Delta z]$ with n is observed for very small n, due to the effect of demographic stochasticity as explained in the main text. Avoiding these small values of n, we applied linear regression to $\mathbb{E}[\Delta z]$ in the small n/N regime and used the intercept (thick black line) as the measured value of E_0.

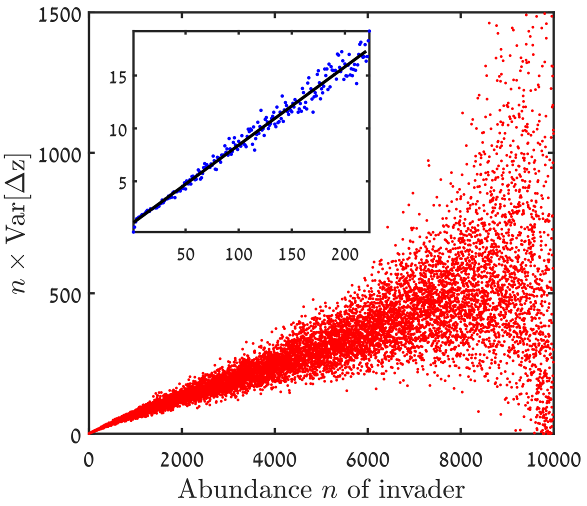

FIG. S16. $n \times \mathrm{Var}[\Delta z]$ vs. n. A linear regression fit at small n values (inset, regression line shown in black) yields V_e as its slope and V_d as its intercept.

S9. THE LIMITS $V_{\mathrm{d}} = 0$ (IN WHICH A THRESHOLD REPLACES DEMOGRAPHIC STOCHASTICITY), AND $V_{\mathrm{e}} = 0$

In this supplement we consider the limits of our formulae when V_{e} (or $\mathcal{V}_{\mathrm{e}}$) vanishes and when the effect of demographic stochasticity is taken into account only as a threshold density below which a population is considered extinct. The first case is simple; the second requires some elaboration.

S9.1. The case of vanishing V_{e}

As explained in the main text, environmental stochasticity has two possible effects: it increases the amplitude of stochastic variations such that the variance of abundance fluctuations per τ-period has a $\mathcal{V}_{\mathrm{e}}n^2$ term, and it may affect (through mechanisms like the storage effect and relative nonlinearity) the value of the mean growth rate. Importantly, these two effects are independent. Consider an extreme case when the environment changes after each elementary step. Then there may still be covariance between competition and environment but there is no $\mathcal{V}_{\mathrm{e}}n^2$ term in the variance. The same is true when the invading species is very rare, so the chance that the environment flips between two birth-death event is large. Therefore, the case $V_{\mathrm{e}} = 0$ includes two scenarios: invasion in a fixed environment and invasion when the environmental variations are fast with respect to the birth-death process. In both these scenarios the chance of invasion is determined by the mean growth parameter and a demographic stochasticity term, without there being any environmental stochasticity term – yet the environmental variations may make their presence felt indirectly, through their influence on the mean growth and the demographic stochasticity.

In either case, when $\mathcal{V}_{\mathrm{e}} \to 0$ the diffusion approximation formula simplifies as follows. Since $\mathcal{R} = \mathcal{V}_{\mathrm{e}}/\mathcal{V}_{\mathrm{d}} \to 0$, the term $(1 + \mathcal{R}n)^{\mathcal{Q}} \approx \exp(n\mathcal{R}\mathcal{Q})$. In this limit $\mathcal{Q} \approx -2\mathcal{E}/\mathcal{V}_{\mathrm{e}}$. Accordingly, $(1 + \mathcal{R}n)^{\mathcal{Q}} \approx \exp(-2\mathcal{E}n/\mathcal{V}_{\mathrm{d}})$. Eq. (6) of the main text thus becomes

$$\Pi^{\mathrm{DA}}_{n\to n_{\mathrm{f}}} \approx \frac{1 - e^{-2\mathcal{E}n/\mathcal{V}_{\mathrm{d}}}}{1 - e^{-2\mathcal{E}n_{\mathrm{f}}/\mathcal{V}_{\mathrm{d}}}}. \tag{S19}$$

A similar argument applies to the WKB-based formula. If $V_{\mathrm{e}} \to 0$ then $\overline{q}$ diverges and therefore R, which is inversely proportional to $\overline{q}$, tends to zero. As a result, $(1 + Rn)^{\overline{q}} \approx \exp(nR\overline{q}) = \exp(-2E_0 n/V_{\mathrm{d}})$, and Eq. (20) of the main text reduces to

$$\Pi^{\mathrm{WKB}}_{n\to n_{\mathrm{f}}} \approx \frac{1 - e^{-2E_0 n/V_{\mathrm{d}}}}{1 - e^{-2E_0 n_{\mathrm{f}}/V_{\mathrm{d}}}}. \tag{S20}$$

These expressions are in agreement with known results that were obtained long ago for the case of a fixed environment using branching process analysis [14] or the diffusion approximation [15]. Our technique makes it possible to obtain

higher-order corrections to these expressions.

S9.2. Replacing demographic stochasticity with absorbing boundary at a given abundance threshold

When the size n of the invading population is large, the abundance variations induced by demographic stochasticity (which scale like $\mathcal{V}_{\rm d} n$) are much weaker than the variations induced by environmental stochasticity (which scale like $\mathcal{V}_{\rm e} n^2$) [16]. Therefore, many theories of community dynamics, permanence, and stability properties ignore demographic stochasticity [17–20].

However, demographic stochasticity reflects the inescapable quantization of the number of individuals in a population. Without demographic stochasticity, even an exponentially decreasing population never reaches zero. As a result, while in reality a population can certainly go extinct, in theories without demographic stochasticity strict extinction is impossible: as its environment varies, a population may always recover from long periods of decline [21]. To account for this problem, theories without demographic stochasticity define a threshold value below which a population is considered extinct [7, 20]. The choice of this threshold is arbitrary. This does not pose a problem for theories of permanence and uniform stability, because these theories are focused on a binary distinction between stable and unstable systems, which does not depend on the exact location of the threshold. However it can be very instructive to make these theories quantitative or semi-quantitative by an informed choice of this threshold. In this subsection we suggest such an appropriate choice by comparing our results with the outcome of a theory without environmental stochasticity.

When demographic stochasticity is neglected, the invading population follows the dynamics of an asymmetric random walk along the z-axis. If, additionally, the diffusion approximation holds (Π_z is smooth enough over z and $E_0^2 \ll V_{\rm e}$), then the chance of invasion satisfies the biased diffusion equation,

$$\frac{V_{\rm e}}{2}\Pi''(z) + E_0\Pi'(z) = 0. \tag{S21}$$

The solution of Eq. (S21), with the boundary conditions $\Pi(z_{\rm th}) = 0$ and $\Pi(z_{\rm f}) = 1$, gives the chance of a random walker at z to reach a given final destination $z_{\rm f} > z$ before reaching some lower threshold value $z_{\rm th} < z$. This solution takes the form [22]

$$\Pi(z) = \frac{1 - e^{-\frac{2\mathbb{E}[\Delta z]}{V_{\rm e}}(z - z_{\rm th})}}{1 - e^{-\frac{2\mathbb{E}[\Delta z]}{V_{\rm e}}(z_{\rm f} - z_{\rm th})}}. \tag{S22}$$

This well-known result was applied to population dynamics in [10] [their Eq. (46)].

How can one obtain Eq. (S22) from Eq. (20) of the main text? When $V_{\rm d} \to 0$, R diverges. Therefore,

$$(1+Rn)^{\overline{q}} = e^{\overline{q}\ln(1+Rn)} \approx e^{\overline{q}(\ln n + \ln R)}. \tag{S23}$$

When the diffusion approximation holds ($E_0^2 \ll V_{\rm e}$), one finds

$$\overline{q} \approx -\frac{2\mathbb{E}[\Delta z]}{V_{\rm e}}, \qquad R \approx \frac{V_{\rm e}}{V_{\rm d}}. \tag{S24}$$

Therefore, $\Pi^{\rm WKB}$ takes the form

$$\Pi_n \approx \frac{1-e^{-\frac{2\mathbb{E}[\Delta z]}{V_{\rm e}}\ln(1+nV_{\rm e}/V_{\rm d})}}{1-e^{-\frac{2\mathbb{E}[\Delta z]}{V_{\rm e}}\ln(1+n_{\rm f}V_{\rm e}/V_{\rm d})}} \approx \frac{1-e^{-\frac{2\mathbb{E}[\Delta z]}{V_{\rm e}}[\ln n - \ln(V_{\rm d}/V_{\rm e})]}}{1-e^{-\frac{2\mathbb{E}[\Delta z]}{V_{\rm e}}[\ln n_{\rm f} - \ln(V_{\rm d}/V_{\rm e})]}}. \tag{S25}$$

Writing $z = \ln n$ and $z_{\rm f} = \ln n_{\rm f}$, one realizes that the two expressions Eq. (S22) and Eq. (S25) coincide if

$$n_{\rm th} = \frac{V_{\rm d}}{V_{\rm e}}. \tag{S26}$$

The correct threshold, thus, appears at the abundance level at which the strength of the demographic stochasticity becomes equal to that of the environmental stochasticity. Note that the derivation of Eqs. (S22) and (S25) assume that the diffusion approximation holds. When this is not the case, our analysis suggests that the threshold has to be taken at $n_{\rm th} \approx 1/R$.

[1] J. Pande and N. M. Shnerb, Taming the diffusion approximation through a controlling-factor WKB method, Physical Review E **102**, 062410 (2020).

[2] D. A. Kessler and N. M. Shnerb, Extinction rates for fluctuation-induced metastabilities: a real-space WKB approach, Journal of Statistical Physics **127**, 861 (2007).

[3] M. Assaf and B. Meerson, Noise enhanced persistence in a biochemical regulatory network with feedback control, Physical Review Letters **100**, 058105 (2008).

[4] M. Assaf and B. Meerson, WKB theory of large deviations in stochastic populations, Journal of Physics A: Mathematical and Theoretical **50**, 263001 (2017).

[5] T. N. Grainger, J. M. Levine, and B. Gilbert, The invasion criterion: A common currency for ecological research, Trends in Ecology & Evolution **34**, 925 (2019).

[6] P. L. Chesson and R. R. Warner, Environmental variability promotes coexistence in lottery competitive systems, The American Naturalist **117**, 923 (1981).

[7] P. L. Chesson, The stabilizing effect of a random environment, Journal of Mathematical Biology **15**, 1 (1982).

[8] J. Pande, T. Fung, R. Chisholm, and N. M. Shnerb, Mean growth rate when rare is not a reliable metric for persistence of species, Ecology Letters **23**, 274 (2020).

[9] M. Danino, N. M. Shnerb, S. Azaele, W. E. Kunin, and D. A. Kessler, The effect of environmental stochasticity on species richness in neutral communities, Journal of Theoretical Biology **409**, 155 (2016).

[10] A. Dean and N. M. Shnerb, Stochasticity-induced stabilization in ecology and evolution: a new synthesis, Ecology **101**, e03098 (2020).

[11] J. Usinowicz, S. J. Wright, and A. R. Ives, Coexistence in tropical forests through asynchronous variation in annual seed production, Ecology **93**, 2073 (2012).

[12] J. Usinowicz, C.-H. Chang-Yang, Y.-Y. Chen, J. S. Clark, C. Fletcher, N. C. Garwood, Z. Hao, J. Johnstone, Y. Lin, M. R. Metz, *et al.*, Temporal coexistence mechanisms contribute to the latitudinal gradient in forest diversity, Nature **550**, 105 (2017).

[13] V. Mustonen and M. Lässig, Molecular evolution under fitness fluctuations, Physical Review Letters **100**, 108101 (2008).

[14] J. B. S. Haldane, A mathematical theory of natural and artificial selection, part v: selection and mutation, in *Mathematical Proceedings of the Cambridge Philosophical Society*, Vol. 23 (Cambridge University Press, 1927) pp. 838–844.

[15] M. Kimura, On the probability of fixation of mutant genes in a population, Genetics **47**, 713 (1962).

[16] R. Lande, S. Engen, and B.-E. Saether, *Stochastic Population Dynamics in Ecology and Conservation* (Oxford University Press, 2003).

[17] P. Schuster, K. Sigmund, and R. Wolff, Dynamical systems under constant organization. iii. cooperative and competitive behavior of hypercycles, Journal of Differential Equations **32**, 357 (1979).

[18] J. Hofbauer, A general cooperation theorem for hypercycles, Monatshefte für Mathematik **91**, 233 (1981).

[19] V. Hutson and K. Schmitt, Permanence and the dynamics of biological systems, Mathematical biosciences **111**, 1 (1992).

[20] S. J. Schreiber, M. Benaïm, and K. A. Atchadé, Persistence in fluctuating environments, Journal of Mathematical Biology **62**, 655 (2011).

[21] A. Szilágyi and G. Meszéna, Coexistence in a fluctuating environment by the effect of relative nonlinearity: a minimal model, Journal of Theoretical Biology **267**, 502 (2010).

[22] S. Redner, *A Guide to First-Passage Processes* (Cambridge University Press, 2001).
